# Optogenetically transduced human ES cell-derived neural progenitors and their neuronal progenies: Phenotypic characterization and responses to optical stimulation

**DOI:** 10.1371/journal.pone.0224846

**Published:** 2019-11-11

**Authors:** Jiwon Ryu, Philippe F. Y. Vincent, Nikolaos K. Ziogas, Leyan Xu, Shirin Sadeghpour, John Curtin, Athanasios S. Alexandris, Nicholas Stewart, Richard Sima, Sascha du Lac, Elisabeth Glowatzki, Vassilis E. Koliatsos

**Affiliations:** 1 Department of Pathology, Johns Hopkins University School of Medicine, Baltimore, Maryland, United States of America; 2 Department of Otolaryngology Head and Neck Surgery, Johns Hopkins University School of Medicine, Baltimore, Maryland, United States of America; 3 Division of Neuropathology, Johns Hopkins University School of Medicine, Baltimore, Maryland, United States of America; 4 Department of Psychiatry, Johns Hopkins University School of Medicine, Baltimore, Maryland, United States of America; University of South Florida, UNITED STATES

## Abstract

Optogenetically engineered human neural progenitors (hNPs) are viewed as promising tools in regenerative neuroscience because they allow the testing of the ability of hNPs to integrate within nervous system of an appropriate host not only structurally, but also functionally based on the responses of their differentiated progenies to light. Here, we transduced H9 embryonic stem cell-derived hNPs with a lentivirus harboring human channelrhodopsin (hChR2) and differentiated them into a forebrain lineage. We extensively characterized the fate and optogenetic functionality of hChR2-hNPs *in vitro* with electrophysiology and immunocytochemistry. We also explored whether the *in vivo* phenotype of ChR2-hNPs conforms to *in vitro* observations by grafting them into the frontal neocortex of rodents and analyzing their survival and neuronal differentiation. Human ChR2-hNPs acquired neuronal phenotypes (TUJ1, MAP2, SMI-312, and synapsin 1 immunoreactivity) *in vitro* after an average of 70 days of coculturing with CD1 astrocytes and progressively displayed both inhibitory and excitatory neurotransmitter signatures by immunocytochemistry and whole-cell patch clamp recording. Three months after transplantation into motor cortex of naïve or injured mice, 60–70% of hChR2-hNPs at the transplantation site expressed TUJ1 and had neuronal cytologies, whereas 60% of cells also expressed ChR2. Transplant-derived neurons extended axons through major commissural and descending tracts and issued synaptophysin^+^ terminals in the claustrum, endopiriform area, and corresponding insular and piriform cortices. There was no apparent difference in engraftment, differentiation, or connectivity patterns between injured and sham subjects. Same trends were observed in a second rodent host, i.e. rat, where we employed longer survival times and found that the majority of grafted hChR2-hNPs differentiated into GABAergic neurons that established dense terminal fields and innervated mostly dendritic profiles in host cortical neurons. In physiological experiments, human ChR2^+^ neurons in culture generated spontaneous action potentials (APs) 100–170 days into differentiation and their firing activity was consistently driven by optical stimulation. Stimulation generated glutamatergic and GABAergic postsynaptic activity in neighboring ChR2^-^ cells, evidence that hChR2-hNP-derived neurons had established functional synaptic connections with other neurons in culture. Light stimulation of hChR2-hNP transplants *in vivo* generated complicated results, in part because of the variable response of the transplants themselves. Our findings show that we can successfully derive hNPs with optogenetic properties that are fully transferrable to their differentiated neuronal progenies. We also show that these progenies have substantial neurotransmitter plasticity *in vitro*, whereas *in vivo* they mostly differentiate into inhibitory GABAergic neurons. Furthermore, neurons derived from hNPs have the capacity of establishing functional synapses with postsynaptic neurons *in vitro*, but this outcome is technically challenging to explore *in vivo*. We propose that optogenetically endowed hNPs hold great promise as tools to explore *de novo* circuit formation in the brain and, in the future, perhaps launch a new generation of neuromodulatory therapies.

## Introduction

Regenerative medicine relies heavily on animal models of disease and on preclinical optimization of regenerative therapies, including therapies based on stem cells and their derivatives [1]. In neurology, the consideration of stem-cell based regenerative strategies is founded on a series of successful preclinical transplantation studies showing that exogenous neural stem cells (NSCs) derived from embryonic neural tissue, embryonic stem (ES) cells, or induced pluripotent stem cells (iPSCs) can successfully engraft, differentiate, and integrate into the preformed adult nervous system [2–10] in a manner largely corresponding to their constitutive differentiation program. The demonstration of structurally mature synapses from stem cell transplants that contact host neurons in the brain and spinal cord [5, 7, 10–12] raises the question whether newly formed synapses are not only structurally complete, but also physiologically active. Functionality of regenerated synapses is important not only for the purpose of conveying system-specific information, but perhaps also for facilitating transsynaptic trophic support [13–15].

Human ES cell-derived neural progenitors (NPs) and iPSCs have been induced to a large number of neuronal or glial fates and have been used in preclinical therapies for various neurological diseases, especially stroke [16–18], neurodegenerative diseases like Parkinson’s and Huntington’s disease and Amyotrophic lateral sclerosis [8, 10, 11, 19–21], and spinal cord injury [22, 23]. Traumatic brain injury (TBI), another common problem with 2–3 million new cases requiring medical attention annually, is often associated with chronic encephalopathies that have no effective treatments and a disappointing record of universally negative clinical trials [24]. In all the above conditions there is widespread disconnection or degradation of brain circuits. Therefore, a key translational issue is whether stem cells and their derivatives can help support or regenerate parts of damaged circuitry. One way to achieve this goal is to transplant NPs with pre-established neuronal differentiation potential and to monitor their circuit-generating effects with physiological strategies. One strategy is the transplantation of optogenetically competent NPs after transduction with channelrhodopsin (ChR2) genes and then monitoring of their ability to form physiologically active circuits with light stimulation. However, despite attempts to create stable ChR2 human ES cell lines to prevent variable transduction and expression efficiency in ES cell-derived neurons, to date, it has been difficult to establish and successfully maintain such lines.

Here we used a non-clonal strategy to generate optogenetically competent human ChR2 (hChR2)-expressing human NPs (hNPs) that we extensively characterized *in vitro* and then tested their differentiation fate *in vivo* after transplantation into the adult rodent CNS (frontal neocortex). We found that these cells differentiate into mature neurons that stably express the optogene and, when transplanted into the cortex of mice and rats, they survive and differentiate into mature neurons with GABAergic phenotypes that innervate proximal and remote limbic, paralimbic and neocortical sites. In physiological experiments *in vitro* (light stimulation of hChR2-hNP-derived neurons), these nerve cells can elicit glutamatergic and GABAergic postsynaptic activity in neighboring neurons via newly matured synapses.

## Materials and methods

### Derivation of hNPs

Because cerebral cortex is highly accessible to transplantation and commonly implicated in disease, including TBI, our differentiation strategy was designed such as to introduce a forebrain fate bias to cells destined for transplantation. Therefore, H9 stem cells were differentiated into forebrain-specific hNPs by inhibiting bone morphogenetic protein (BMP)-mediated Spinal Muscular Atrophy and Mothers Against Decapentaplegic (SMAD) signaling with dorsomorphin and also epithelial-to-mesenchymal transition with the TGFβ kinase/activin receptor like kinase (ALK)-5 inhibitor A-83-01 (targeting TGF-β type I receptor ALK5, Activin/Nodal receptor ALK4, and nodal receptor ALK7) [25, 26]. Cells were maintained as colonies on mouse embryonic fibroblast (MEF) plates [27, 28] and lifted with collagenase treatment (37°C, 1 hour) to generate embryoid bodies (EB) on Day 0. Embryoid bodies were fed daily with EB medium containing DMEM/F12, 20% KOSR, 2mM L-glutamine, 0.1mM NEM-NEAA, 100μM 2-mercaptoethanol, 2μM dorsomorphin and 2μM A-83 for 4 days and then human NP medium (DMEM/F12, 1× N2, 0.1mM NEM-NEAA, 2μg/mL heparin, 2μM cyclopamine) from day 5 to 21 every two days. On day 7, EBs were plated on Matrigel coated plates and the resulting rosettes were collected manually with pipette on day 22 to generate neurospheres in human NP medium. To expand NPs, spheres were digested with accutase and 1–2 million cells were plated on matrigel-coated 35mm dishes and fed with NP expansion medium containing DMEM/F12, 1× N2, 1× B27 and 20ng/mL FGF2. Cells were characterized at different stages of differentiation with immunocytochemistry (ICC) for OCT4, nestin, PAX6, FOXG1, HB9, TUJ1, MAP2 and SMI-312.

### Transduction of human NPs with lentivirus harboring hSyn-hChR2

Our early attempts to establish a stable hChR2 transgenic human embryonic stem cell (hES) clonal line to avoid variable transduction and expression efficiency was hampered by problems in stable transgene expression despite the presence of the transgenes hChR2 and YFP in the genomic DNA (S1 Fig and S1 Table). After differentiation of H9 cells to NPs and neurons, transgene expression was not detectable; varying neural differentiation methods. Therefore, we decided to transduce cells at the NP stage of differentiation. Neural progenitor cells (2 million) were plated on a matrigel-coated 35mm dish on day 34. Two days after plating, cells were treated with polybrene (6 μg/mL) in NP expansion medium at 37°C for 15min. Lentivirus harboring hSyn-hChR2(H134R)-eYFP-WPRE was added to each well at moi 0 or 13 and incubated at 37°C for 24 hours. The virus medium was replaced with new NP expansion media (NPEM; DMEM/F12, N2, B27, 20ng/mL FGF2) on the next day and live cell images of NPs or neurons expressing hChR2 were acquired 4–5 days after transduction using a Nikon TS 100 fluorescence inverted microscope (Nikon Instruments Inc., Melville, NY) with a Digital Sight DS Fi1 CCD camera (Nikon Instruments Inc., Melville, NY).

### Differentiation to neurons with forebrain signatures

Human ChR2-NPs used for transplantation at day 41 were subjected to further *in vitro* differentiation to neurons with a forebrain phenotype, essentially as described in Wen et al, 2014 [26]. Neural progenitors were plated on matrigel-, laminin- or PDL/laminin-coated chambers (ibidi USA Inc., Madison, Wisconsin; 8 well, 80841) or 12 mm coverslips (Bellco Glass Inc., Vineland, NJ; 1943-10012A or Corning, Corning, NY; Biocoat 354087) for further forebrain-specific differentiation on day 41 and were fed with neural differentiation media (NDM) containing Neurobasal medium, 2mM L-glutamine, 1× B27, 10ng/mL BDNF and 10ng/mL GDNF every 2–3 days [26]. Neurons were characterized by immunocytochemistry for NP markers (PAX6, FOXG1, BRN2, and TBR1), neuronal markers (TUJ1, MAP2, SMI-312, Synapsin 1, GAD1, vGLUT1/2 and vGAT) and astrocytic markers (GFAP) as described [27, 28]. Donkey anti-rabbit or mouse IgG-Alexa-Fluor 488, 555, 655, donkey anti-chicken IgY-Alexa-Fluor 488 and donkey anti-mouse IgG-Cy3 were used as secondary antibodies at concentrations of 1:500–1:1000.

### Mouse primary astrocyte preparation for co-culture with hNPs and neurons

Primary astrocytes were prepared from P2-P3 CD1 mouse brains as described [29]. Twenty-to-forty thousand astrocytes were plated on 12 mm coverslips coated with poly-D-lysine (PDL) and laminin a week before hNP plating and fed with astrocyte progenitor medium containing DMEM (Invitrogen, Carlsbad, CA, 10569), FBS-HI (Invitrogen, 10082) and penicillin/streptomycin (Invitrogen, 15140). Twenty to eighty thousand hNPs (day 41–50) were plated on the coverslips and fed with NDM every 2–3 days to promote neuronal differentiation for electrophysiology. Human ChR2-neurons expressing YFP were used for *in vitro* optogenetic stimulation and testing of their response to light at a 485 nm wavelength.

### Whole-cell patch clamp recordings and data analysis

Cultured neurons were placed in a recording chamber under a Zeiss Axioskop 2 FS plus or a Nikon A1R-MP upright microscope and visualized with a 40× water immersion objective. Cells were perfused at a rate of 1mL/min with an extracellular solution containing (in mM): 140 NaCl, 3 KCl, 2 CaCl_2_, 1 MgCl_2_, 15 HEPES, 23 glucose, pH 7.4 (NaOH), 300 mOsm. For some experiments, 250 nM TTX citrate (Tocris, Minneapolis, MN) was added to the extracellular solution to block voltage-gated sodium channels. Borosilicate patch pipettes with 1 mm outside diameter (WPI, Sarasota, FL) were pulled using a multistep horizontal puller P87 (Sutter Instrument, Novato, CA) and fire polished with a micro forge MF-200 (WPI) to achieve pipette resistances ranging from 4 to 5 MΩ. Patch pipettes were filled with an intracellular solution containing (in mM): 121 K-gluconate, 22 KCl, 10 HEPES, 10 EGTA, 4 Mg-ATP, pH 7.2 (KOH), 290 mOsm. All recordings were performed at room temperature (21–24°C).

Data were either collected using pClamp10.6 software with a Multiclamp 700B amplifier (Molecular Devices, Sunnyvale, CA) or using patchmaster controlling a double EPC10 Heka amplifier (Heka Elektronik, Germany). The signal was low-pass filtered at 10 kHz and digitized at 20 kHz with a Digidata 1550A (Molecular Devices). Only recordings with series resistances (Rs) < 25 MΩ and leak currents < 200 pA were considered for analysis, without compensating for Rs errors. In voltage clamp mode, holding voltage potentials were corrected for liquid junction potentials (11 mV).

Data were analyzed using Clampfit 10.6 (Molecular Devices), MiniAnalysis (Synaptosoft, Fort Lee, NJ) and Origin 9.1 (OriginLab, Northampton, MA). Statistical testing was performed with Origin 9.1 software. When comparing two independent samples, an unpaired student t-test was used. In the case of two dependent samples, a paired student t-test was used. Where more than two groups were compared, ANOVA was used. The limit of significance was set at p < 0.05.

### *In vitro* optical stimulation

Channelrhodopsin activation was achieved with a custom LED device including an optic fiber cable mounted on a 485 nm blue LED (Cree Lighting Inc., Durham, NC) connected to a SLB-1200-1 universal LED driver (Mightex, Pleasanton, CA). The end of the optic fiber cable was placed a few millimeters away from the cells with a MP-285 micromanipulator (Sutter Instrument, Novato, CA). Light intensity was calibrated at 0.38 mW/mm^2^ using a PM20A optical power meter (Thorlabs, Newton, NJ). Light pulses were Transistor-Transistor Logic (TTL) triggered with pClamp10.6 software using the analog-input of the LED driver. For some sets of experiments, channelrhodopsin was stimulated using a Spectra X (Lumencor, Beaverton, USA) directly coupled to the epifluorescent port of the Nikon A1R-MP microscope. Light pulses were TTL triggered with patchmaster software.

### Transplantation of hChR2-hNPs in the frontal cortex of mice and rats

Fourteen-to-fifteen-week old male athymic nude mice (Hsd:Athymic Nude-Foxn1^nu^; Envigo, Indianapolis, IN) and 12 weeks old athymic nude rats (Crl:NIH-Foxn1^rnu^) were the subjects of transplantation experiments that were carried out according to protocols approved by the Animal Care and Use Committee of the Johns Hopkins Medical Institutions. Immunodeficient rodents allow for a better engraftment and survival of human cells compared to immunocompetent animals treated with immunosuppressants [30]. Transplantation was performed using gas anesthesia (isoflurane:oxygen:nitrous oxide = 1:33:66) and aseptic methods. In mice, we explored the survival and differentiation of both transduced and nontransduced transplanted hNPs in naïve or injured animals (transduced NP, *n* = 45; nontransduced NP, *n* = 4). For injury, we used the impact acceleration method [31] that produces diffuse trauma, with impact set at 40 × 1m [32]. A hundred thousand hNPs were transplanted next to the corpus callosum using pulled beveled glass micropipettes with tip diameters of 50–100μm controlled by a Nanoinjector device (World Precision Instruments, Sarasota, FL) [5, 7, 9, 28]. Stereotaxic coordinates were 0.7 mm anterior to bregma, 1.5 mm lateral to midline and 2 mm ventral to pia. Prior to transplantation, day 41 hNP cells had been dissociated with accutase (Invitrogen) and resuspended in NP expansion media (DMEM/F12, N2, B27, 20ng/mL FGF2) at a concentration of 100,000 per μL. Injured subjects were grafted one week after injury, i.e. time point that appears to optimize survival and differentiation [9, 10]. Animals were allowed to survive for 2–4 months, i.e. a period sufficient to ascertain the success of engraftment and differentiation, including the stable expression of hChR2 *in vivo*. Similarly, naïve athymic rats (uninjured, n = 9) were injected with 50K-100K of hChR2-hNPs into layers 2–3 of the motor cortex (1 mm anterior to bregma, 2.5 mm lateral to midline and 1 mm ventral to pia) using 5 μL Hamilton syringe. Rats were allowed to survive for 9 months before analyzed.

### Histology, immunohistochemistry and microscopy

Brain tissues were prepared from animals perfused transcardially with 4% phosphate-buffered paraformaldehyde as described [5]. The survival and fate of hNP grafts were assessed with avidin-biotin complex-based peroxidase immunohistochemistry (IHC) and dual-label fluorescent IHC in serial coronal sections (40μm) through the brain as described previously [5, 11, 33]. Human NP survival was studied with human-specific nuclei (HuNu) or human-specific cytoplasm (SC121) antibody using immunoperoxidase or immunofluorescence labeling. Differentiation was studied with dual-label immunofluorescence combining antibodies against HuNu or SC121 with antibodies against the neural and glial markers doublecortin, TUJ1, PDGFRα, GFAP, MAP2, and synaptophysin, vGAT, vGLUT1, vGLUT2, GAD1, gamma aminobutyric acid (GABA), oligodendrocyte transcription factor 2 (Olig2), myelin basic protein (MBP), 2',3'-cyclic-nucleotide 3'-phosphodiesterase (CNPase); the mesodermal lineage marker BMP4 or antibodies against YFP; the immediate early gene transcription marker c-FOS as a marker of neuronal activation; and the nuclear epitope Ki67 present in all phases of cell cycle except G0 as a marker of dividing neurons [2, 5, 10, 11]. Information for all antibodies is listed in **S2 Table**. For immunofluorescence, brains were first incubated in the primary antibodies overnight at 4°C, and then incubated in donkey/goat anti-rabbit IgG conjugated to Alexa-Fluor 488, 546 or 647 (1:300, Invitrogen, Carlsbad, CA), donkey/goat anti-mouse IgG conjugated to Cy3 (1:200; Jackson ImmunoResearch, West Grove, PA) or Alexa-Fluor 488, goat anti-guinea pig IgG Alexa Fluor 568 (Abcam, Cambridge, MA) and donkey/goat anti-chicken IgY conjugated to Alexa-Fluor 488 (1:300, Invitrogen, Carlsbad, CA) for 2 hours at 4°C. Brains were then counterstained with the fluorescent DNA dye 4′,6-diamidino-2-phenylindole (DAPI), and then mounted and coverslipped. For the immunoperoxidase-DAB method, after the primary antibody reaction (4°C, overnight), sections were incubated with biotinylated donkey antimouse IgG (1:200; Jackson ImmunoResearch, West Grove, PA) (room temperature, 2 hours), then treated with avidin-HRP and developed in DAB (Vectastain Elite ABC Kit, Vector Laboratories Inc., Burlingame, CA). Stained brain sections were imaged on a Zeiss Axiophot microscope equipped for epifluorescence or a Zeiss LSM 710 confocal microscope, or Leica SP8 confocal microscope with MetaXpress high-content analysis software (Molecular Devices, CA, USA) or Leica Application Suite X (Leica microsystem, Inc, IL). Adobe Photoshop 9.0 software (Adobe Systems, San Jose, CA) and Image J (Fiji distribution) and Imaris 9.0 (Bitplane Inc, Concord, MA) was used for image processing and montaging.

### Stereological estimates of transplanted hChR2-hNP survival and differentiation

In mice transplanted with ChR2-hNPs, SC121-, YFP-, HuNu- and TUJ1-immunoreactive cells were counted through the site of the main transplant and sites of leakage in the ventricle or surface of the brain (pia) on systematically sampled sections (every 6th of a coronal brain series) using ImageJ software (V. 1.44p, NIH, USA). Counting frame was set at 250×250μm per image. The percentage of eYFP^+^SC121^+^ or TUJ1^+^ HuNu^+^ cells was calculated by dividing the number of cells with the total of SC121^+^ or HuNu^+^ cell profiles, respectively.

Student's t-tests were performed to compare between densities of TUJ1^+^ human-derived cells in cortex between transduced and nontransduced NPs at the main transplantation site. One-way analysis of variance (ANOVA) followed by Tukey's post-hoc test was also used to compare the effect of engraftment site (main transplant, pia, ventricles) on NP differentiation based on TUJ1 phenotype (TUJ1^+^HuNu^+^ cells divided by the total of HuNu^+^ cells). Statistical analyses were performed with STATISTICA 8.0 software (StatSoft Inc., Tulsa, OK).

## Results

### Embryonic stem cell differentiation to hNPs with forebrain fate preference

**Fig 1:**
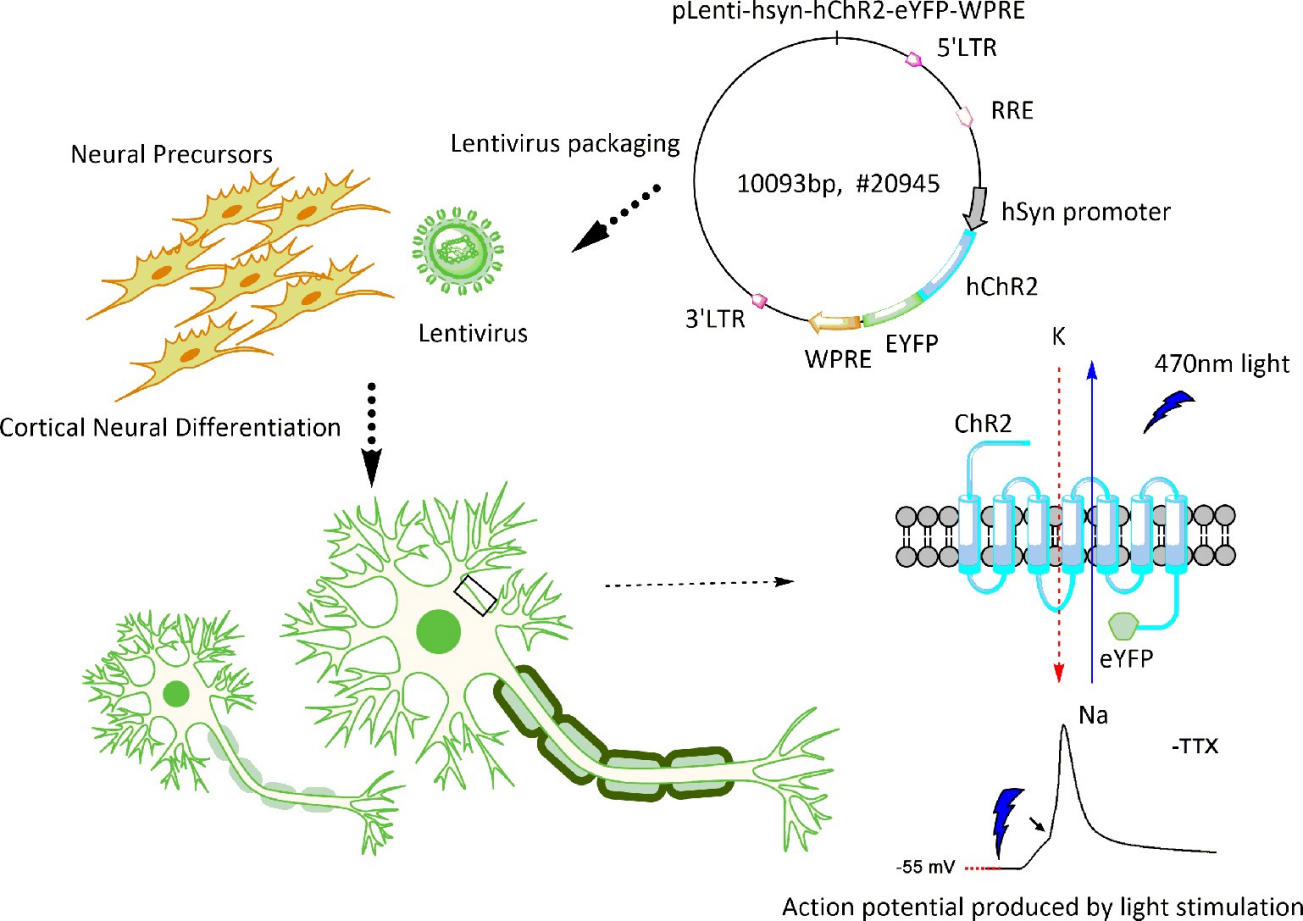
General schema of hES cell differentiation. Optogenetically engineered human neural precursors were differentiated into neurons with forebrain fate preference. The functionality of derived neurons was measured by their light-responsive action potentials and synaptic activity *in vitro* and the induction of the immediate early gene c-FOS in host neurons *in vivo*.

**Fig 2:**
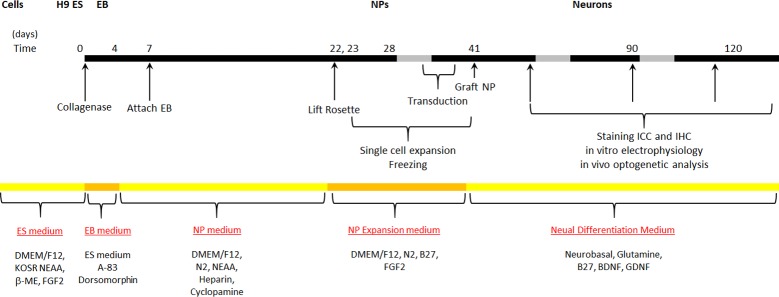
Induction and differentiation protocol used to generate hNPs from H9 hES cells and to further advance them to a cortical neuronal fate. ES, embryonic stem cell; KOSR, KnockOut^™^ Serum Replacement; NEAA, non-essential amino acids; 2-ME, 2-mercaptoethanol; EB, embryoid body; NP, neural progenitors; FGF, fibroblast growth factor 2; BDNF, brain-derived neurotrophic factor; GDNF, glial cell-derived neurotrophic factor.

**Fig 3:**
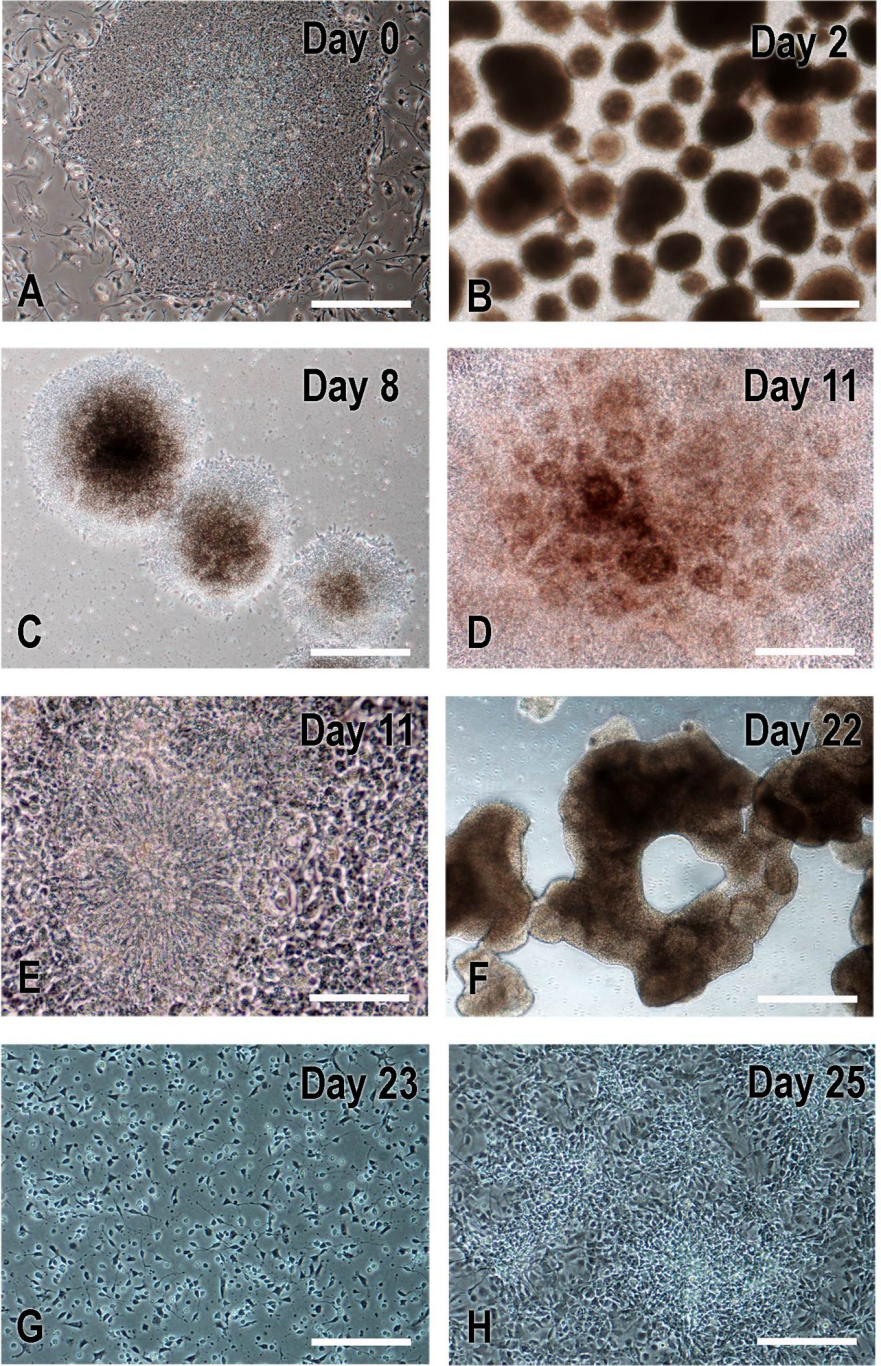
Critical steps in neural differentiation of human ES cells (Day 0) to NPs (Day 25). On Day 0, H9 ES cell colonies (A) were detached using collagenase to form embryoid bodies (B; EBs on Day 2) that were further cultured in EB media with dorsomorphin and A-83. Beginning at day 4, medium was changed to the one promoting NP cell differentiation that was used to feed every other day to Day 22. On Day 7, EBs were attached to matrigel-coated plate; neural rosette structures were evident at day 8 (C) and became prominent on day 11 (D-E). On day 22, colonies with rosettes were detached manually to form neurospheres (F). On day 23, neurospheres were dissociated into single cells with accutase, plated on matrigel-coated plate, and fed with NP expansion media till they became confluent (G-H). Cells were passed every 4–5 days. Days 36 NPs were used for viral transduction and Day 41 NPs for transplantation. Scale bars: A-C, F, 500μm; D and G-H, 200μm; E, 100μm.

Embryonic stem cells were first differentiated to cortical-fate hNPs (Figs 1 and 2). Cells grown as colonies on MEF feeder cell layers were detached by collagenase on day 0 to make EBs (Figs 2 and 3) and fed with EB medium daily for 4 days in an ultra-low attachment plate. Embryoid bodies were initially treated with the AMP-activated protein kinase (AMPK) inhibitor dorsormorphin and the ALK inhibitor A-83-1. On day 5, medium was switched to NP-induction medium that was changed every 2 days to day 21. To pattern cells to forebrain fates, we added heparin for the activation of wnt signaling [34, 35] and cyclopamine for inhibition of SHH signaling [36, 37]. On day 7, EBs were seen to be attached to Matrigel-coated plates and, on the next day (day 8), neural rosette structures started to appear on attached colonies (**Fig 3C**). On day 22, rosettes were lifted to generate neurospheres in NP medium (**Fig 3F**) and then neurospheres were dissociated into single cells with accutase and 1–2 million cells were plated in NP expansion medium on Matrigel-coated wells on day 23 **(Fig 3G and 3F)** for cell expansion.

**Fig 4:**
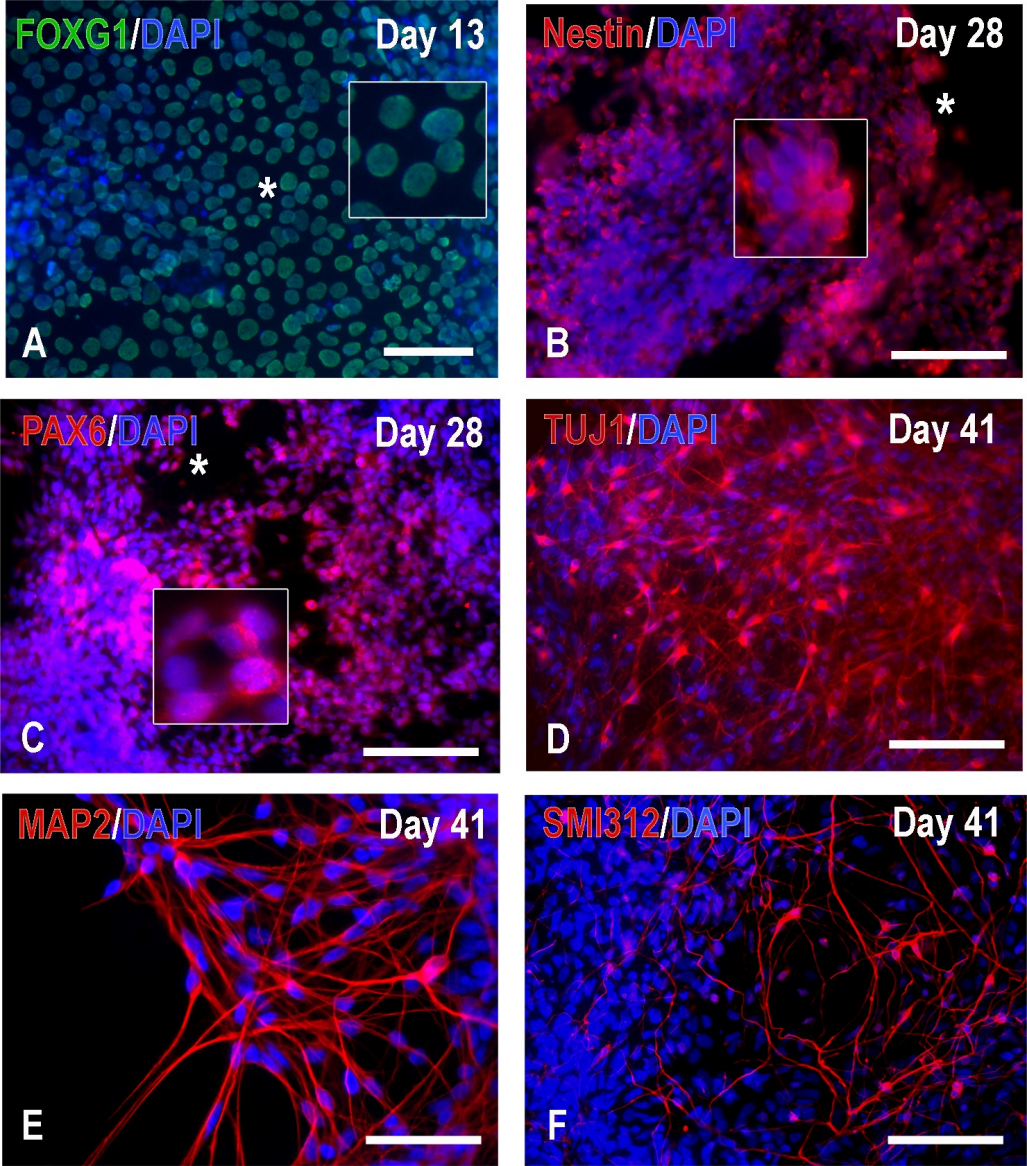
Characterization of H9-derived hNPs by immunocytochemistry at an early stage. Neuronal progenitors were plated on matrigel-coated ibidi chambers or PDL/Laminin coated-12 mm coverslips on day 23. Cells were fed with NDM every 2 days. At Days 13–28, most H9-derived cells expressed the primary progenitor and telencephalon marker FOXG1 (A) and the neural stem cell markers nestin (B) and PAX6 (C); cells immunoreactive for the stem cell marker Oct4 were rare (data not shown). On Day 41 (E-F), some cells were TUJ1, MAP2 and SMI-312 positive but vGAT, vGLUT1 and synapsin 1 signals were absent (data not shown). Scale bars: A, 25μm; B-D and F, 100μm; E, 50μm.

To examine the progressive differentiation of hNPs *in vitro*, on day 23, cells were also plated on coverslips or a plastic surface coated with Matrigel or PDL and laminin at various densities and fed with neural differentiation media every 2–3 days for immunocytochemistry. Consistent with the forebrain neural differentiation method used here [26], the majority of NPs were FOXG1 positive on day 13 **(Fig 4A)**; by day 28, they lost Oct4 expression (data not shown) but were nestin as well as PAX6 positive **(Fig 4B and 4C)** and many displayed early neuronal phenotypes **(Fig 4D)**. By day 41, we observed further differentiation to MAP2^+^ and SMI-312^+^ neurons **(Fig 4E and 4F)**.

### Human NP transduction with hChR2-eYFP-harboring lentivirus; establishment of mature neuronal phenotypes of hChR2-hNPs *in vitro*

**Fig 5:**
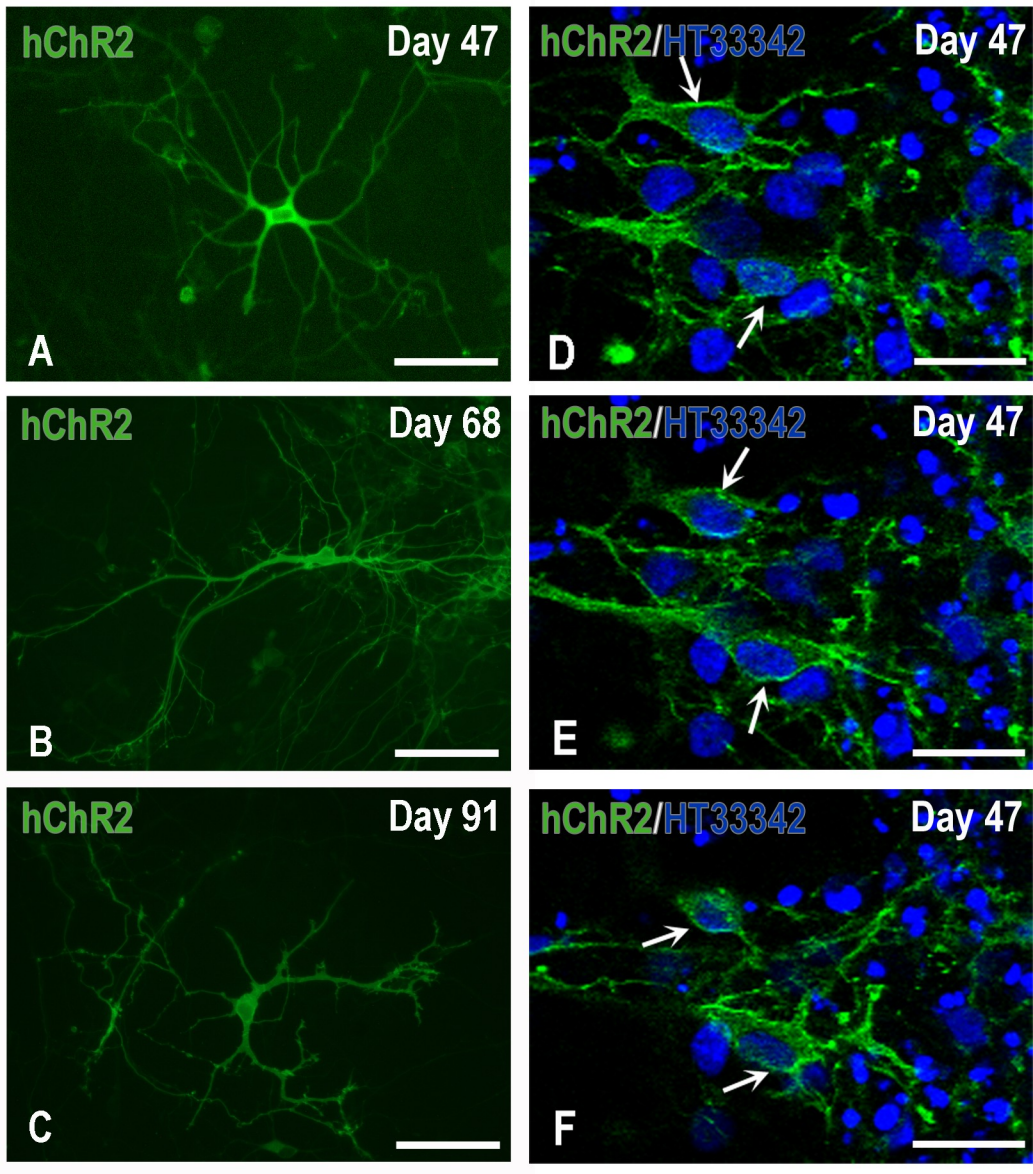
Expression and localization of hChR2 in H9-derived hNPs. Panels A-C depict typical neurons at various days of maturation *in vitro*: H9 hNPs, photographed here on day 47 in culture, had been transduced on Day 34 by lentivirus carrying hChR2 at moi 13. Human ChR2-YFP expression started to appear two-four days after transduction and progressively spread over the perikaryon and processes of maturing neurons. Panels D-F demonstrate the fine localization of hChR2 (YFP fluorescence), predominantly to the membranes of representative transduced nerve cells (arrows), seven days after transduction, by confocal microscopy. Scale bars: A-C, 100μm; D-F, 50μm.

**Fig 6:**
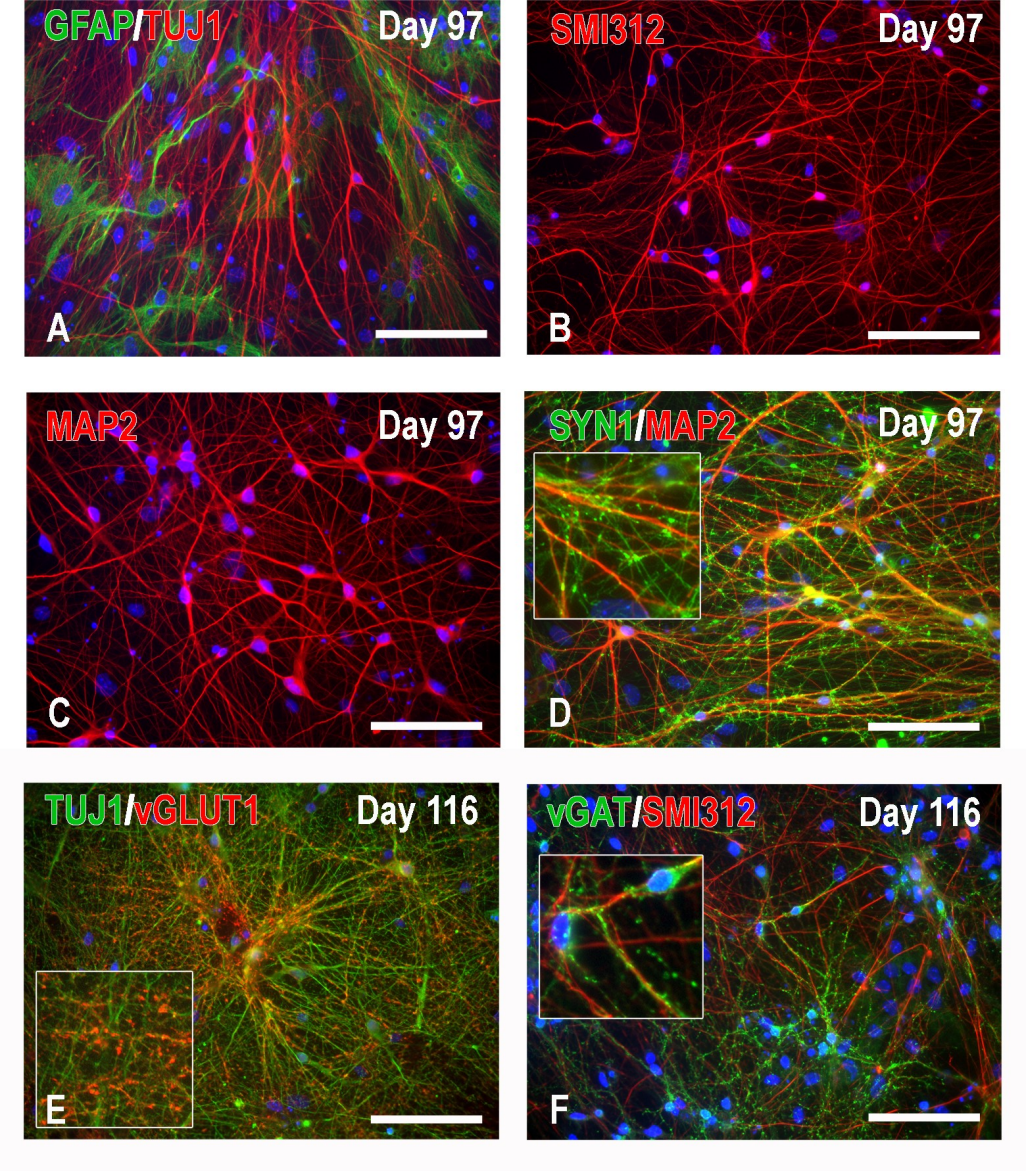
Characterization of hNP-derived neurons co-cultured with mouse astrocytes by immunocytochemistry at an advanced stage (Days 97 and 116). The use of the co-culture system with CD1 astrocytes (A) greatly reduces aggregation and promotes neuronal differentiation in the form of nicely separated mature neurons expressing advanced neuronal signatures such as phosphorylated neurofilaments M and H (B: SMI312 immunoreactivity), MAP2 (C), and SYN1 (D, in conjunction with MAP2). Neurotransmitter markers consistent with excitatory or inhibitory neurotransmission such as vGLUT1 and vGAT are also abundantly expressed in these cultures (E and F). Note the vesicular nature of vGLUT1 and vGAT immunoreactivity. Scale bars: 100μm.

At day 35, some batches of hNPs were transduced with lentivirus at moi of 13 for 24 hours in the presence of polybrene. After transduction, we continued differentiation on PDL- and laminin-coated 12mm coverslips pre-plated with CD1 mouse astrocytes a week prior. Human ChR2 gene expression was detected 4–7 days after transduction **(Fig 5A–5C)**. Confocal microscopy shows that hChR2 protein was localized preferentially at the cell membrane **(Fig 5D–5F)**. Co-culturing hNPs with astrocytes reduced aggregation and inhibition of neuronal maturation that is common when cells are differentiated in NDM long term and led to the appearance of nicely separated mature neurons suitable for phenotypic characterization by microscopy and electrophysiology **(Fig 6A)**. By day 90, neurons co-cultured with astrocytes were seen to express mature neuronal markers including MAP2, SMI-312, synapsin 1 **(Fig 6B–6D)**, and also neurotransmitter markers consistent with inhibitory or excitatory neurotransmission i.e. vGLUT1 for glutamatergic neurotransmission and vGAT for inhibitory GABAergic or glycinergic neurotransmission **(Fig 6E and 6F)**. These neurotransmitter markers display vesicular immunoreactivity **(Fig 6E and 6F)** and colocalize with the endogenous ChR2-YFP (**S2 Fig**).

Next we determined whether hCHR2-expressing nerve cells displayed electrophysiological features of functional neurons and if they formed functional synapses *in vitro*. Human ChR2^+^ cells were kept in co-culture with mouse astrocytes and were identified in the recording dish by their fluorescence (**S3A Fig**). At 6 weeks into co-culturing (corresponding to day 82) hNP-derived neurons were still immature with depolarized resting membrane potentials and weak membrane depolarization in response to optic stimulation. However, at 8–11 weeks, 14 to 17-week old hChR2^+^ neurons were mature enough to allow for the detection of action potentials (APs) and optic responsiveness. Sodium and potassium ion channels were identified in the current voltage relation (**S3B Fig**), and in current clamp mode, spontaneous action potentials (APs) were recorded (**S3C Fig**).

**Fig 7:**
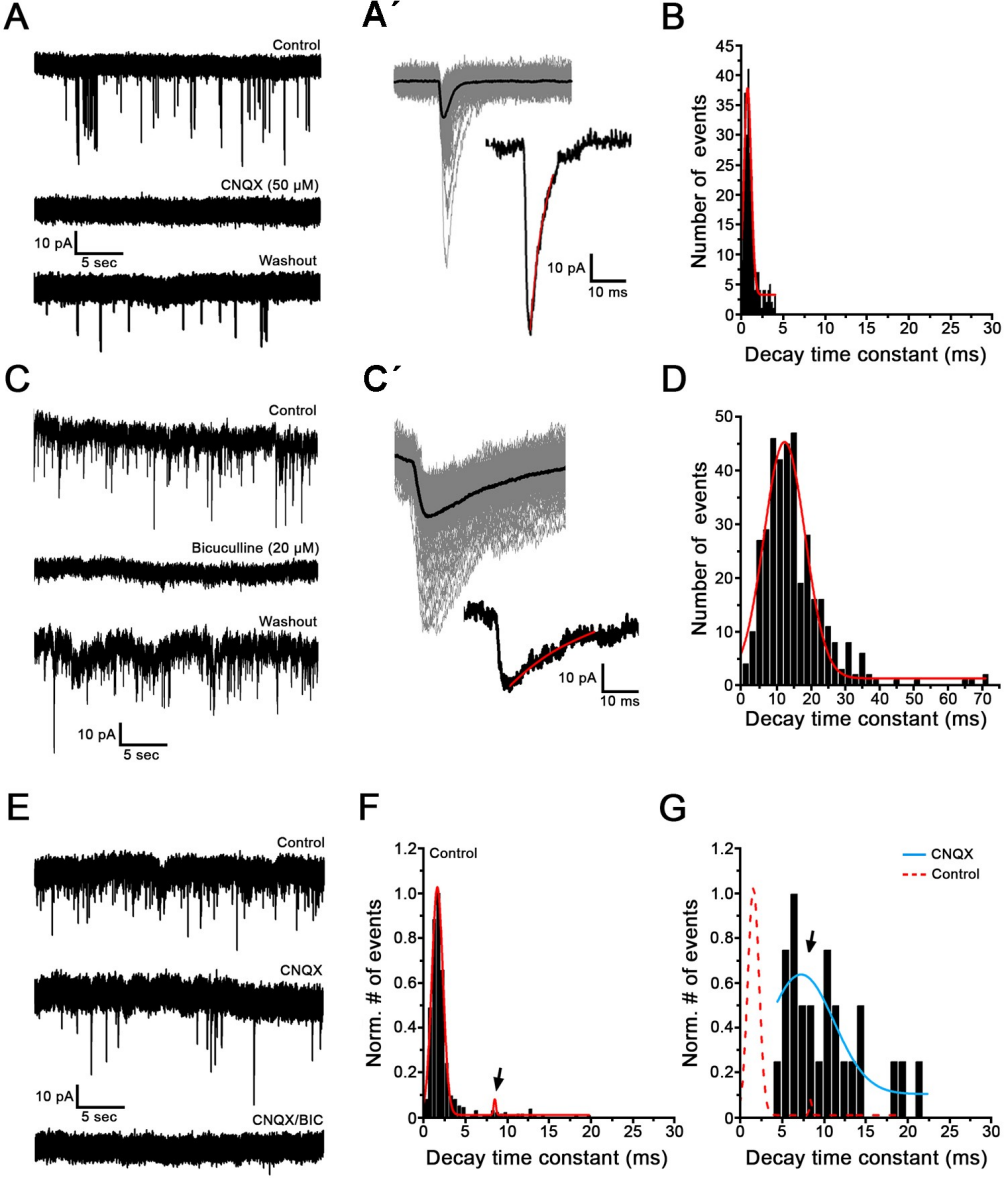
Human NPs display glutamatergic and GABAergic spontaneous synaptic transmission *in vitro* (Days 90–167). Voltage clamp recordings of spontaneous synaptic currents from ChR2^-^ neurons. Holding potential -90 mV; at room temperature. (A) Example recording showing that in this cell, spontaneous synaptic currents are completely and reversibly blocked by 50 μM CNQX, a glutamate receptor blocker. (Aʹ) Top left panel shows a superimposed view of synaptic events from recording in A at an enlarged time scale (gray traces, n = 122 events). Average trace is shown in black. Bottom right panel shows an example of one postsynaptic event. Red line indicates monoexponential fit used to calculate the decay time constant. (B) Distribution of decay time constants for the recording shown in A, with a peak of the distribution at 0.79 ms. (C) Example recording showing that in this cell, spontaneous synaptic currents are completely and reversibly blocked by 20 μM Bicuculline, a GABA-A receptor blocker. (C´) Postsynaptic events from recording in C (n = 131 events, gray lines) were superimposed and averaged (black trace, top left). Bottom right panel shows an example of one postsynaptic current with monoexponential fit of decay time constant. (D) Distribution of decay time constants of the recording shown in C, with a peak of the distribution at 15 ms. Note that events at these inhibitory synapses appear as excitatory postsynaptic currents in this type of recording, as the reversal potential of chloride has been changed in the artificial *in vitro* recording environment. (E) Example recording showing a mix of glutamatergic and GABAergic synaptic events during spontaneous activity. In this cell, CNQX only partially abolished postsynaptic events (middle trace). The remaining events are blocked by bicuculline (bottom trace). (F) Distribution of decay time constants of postsynaptic events from the control trace before drug application in E shows a distribution with two peaks, at 1.6 ms and 8.5 ms (arrow). (G) Distribution of decay time constants of postsynaptic events during CNQX application for the recording shown in the middle trace in E. Note that compared to the distribution in F, the peak at 1.6 ms is abolished in CNQX (red dotted line); therefore it is representing the glutamatergic postsynaptic events. The blue line represents the events that are not blocked by CNQX, but sensitive to bicuculline, and therefore GABAergic. The arrow points to the same peak as in **F**, now appearing larger due to rescaling.

Because immunocytochemical experiments showed excitatory and inhibitory synaptic markers in hNP-derived neurons by day 90, we proceeded to test the presence of functional inhibitory and excitatory synapses with patch clamp recordings from hChR2^-^ neurons between day 90 and day 167. Seventy eight % of recorded neurons (36/46) displayed spontaneous excitatory synaptic currents (EPSCs) **(Fig 7)**. Twenty nine neurons were tested for their transmitter identity, using pharmacology and excitatory postsynaptic current (EPSC) waveform analysis. Fourteen % of neurons (4/29) received uniformly glutamatergic transmission, as their spontaneous EPSCs were completely blocked by 50 μM CNQX, a glutamate receptor blocker specific for AMPA and kainate, but not NMDA receptors **(Fig 7A)**. The blocking effect of CNQX was reversed during washout in 1 out of 4 cells. Excitatory postsynaptic currents were short, as it is typical for AMPA receptor currents. The peak of the distribution for decay time constants was 2.4 ± 1.3 ms (n = 23) **(Fig 7A´ and 7B)**. Seventeen % of neurons (5/29) received uniformly GABAergic transmission, as their spontaneous EPSCs were completely blocked by 20 μM bicuculline, a GABA-A receptor blocker **(Fig 7C)**. The GABAergic block was reversed during washout in 2 out 5 cells. Compared to glutamatergic EPSCs, bicuculline-sensitive EPSCs displayed slower waveforms. The peak of the distribution for decay time constants was 13.1 ± 4.9 ms (n = 24) **(Fig 7C´ and 7D)**. Finally, 69% of cells (20/29) showed mixed synaptic events that were sensitive to either CNQX or to bicuculline block **(Fig 7E)**. For these recordings, the decay time constant distributions displayed two components, representing the faster and slower kinetics (arrow) of the respective glutamatergic and GABAergic EPSCs **(Fig 7F)**. When adding CNQX, fast synaptic events were abolished **(Fig 7G)**, leaving the component that represents the slower kinetics (arrow) of the GABAergic events (n = 3) **(Fig 7G)**. Spontaneous synaptic currents were also recorded from hChR2^+^ neurons in 6 of 8 cells tested. Four recordings had enough EPSCs to be analyzed, and two showed purely glutamatergic and the other two showed purely GABAergic synaptic transmission (data not shown).

Overall, these results suggest that *in vitro*, ChR2^-^ and ChR2^+^ hNPs mature properly into neurons that form a functional network with both excitatory and inhibitory synapses *in vitro*.

### *In vivo* plasticity of hChR2-hNPs: survival and differentiation in mice and rats

Both transduced and non-transduced hNPs were transplanted into the deep sensorimotor cortex (M1-S1) of naïve or injured mice and naïve rats. Mouse and rat material had some key differences. Mice had deeper injections into motor cortex and were allowed to survive only 3 months, to generate observations on the early differentiation of the graft. Rats were intended for optogenetic stimulation experiments *in vivo* because their larger brain size allows for a better spatial separation of transplant site, i.e. the direct target of optic stimulation, and terminal fields of transplant-derived neurons. Also, to readily access and stimulate the transplants from the surface of the brain, and because of the exponential attenuation of energy as the light travels through deeper parts of the brain, we targeted grafting to layers 1–3 of motor cortex. Furthermore, to allow for a full maturation of synapses of hNP-derived neurons, we extended survival time to 9 months, in keeping with our previous observations on the synaptic maturation of NP grafts in the brain [5].

**Fig 8:**
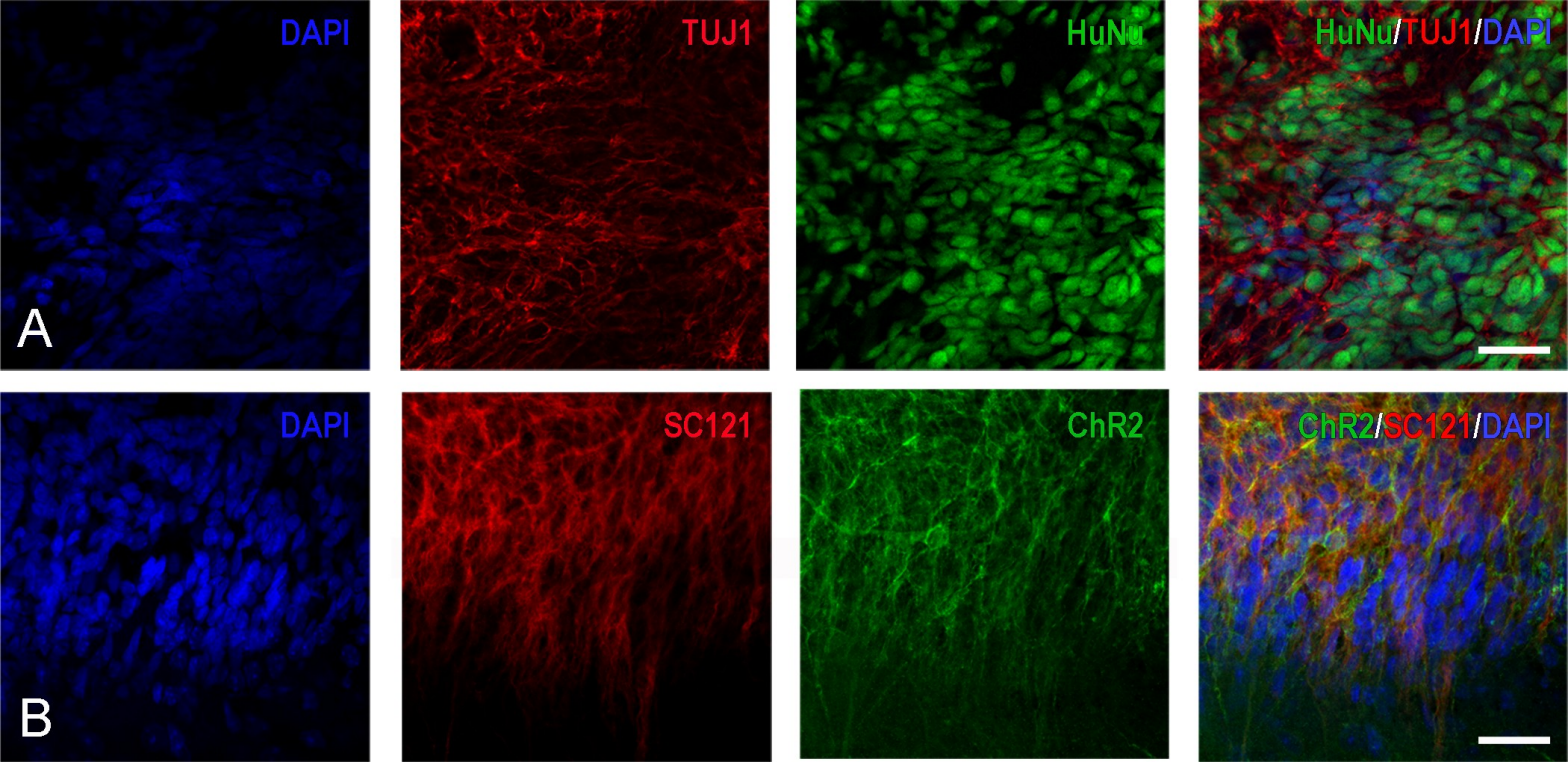
Series of confocal images indicating the differentiation of transplant-derived cells into early neurons with hChR2 expression. (A) These four panels indicate that a large percentage of transplant-derived HuNu^+^ cells (green) are also immunoreactive with antibodies against class III β-tubulin epitope TUJ1 (red). Blue nuclei are stained with DAPI. (B) These panels indicate that the majority of transplant-derived epitope SC121^+^ positive cells (red) also fluoresce in the green spectrum, which is indication of hChR2 expression. Cell nuclei are stained with DAPI (blue). Scale bars: 200μm.

**Fig 9:**
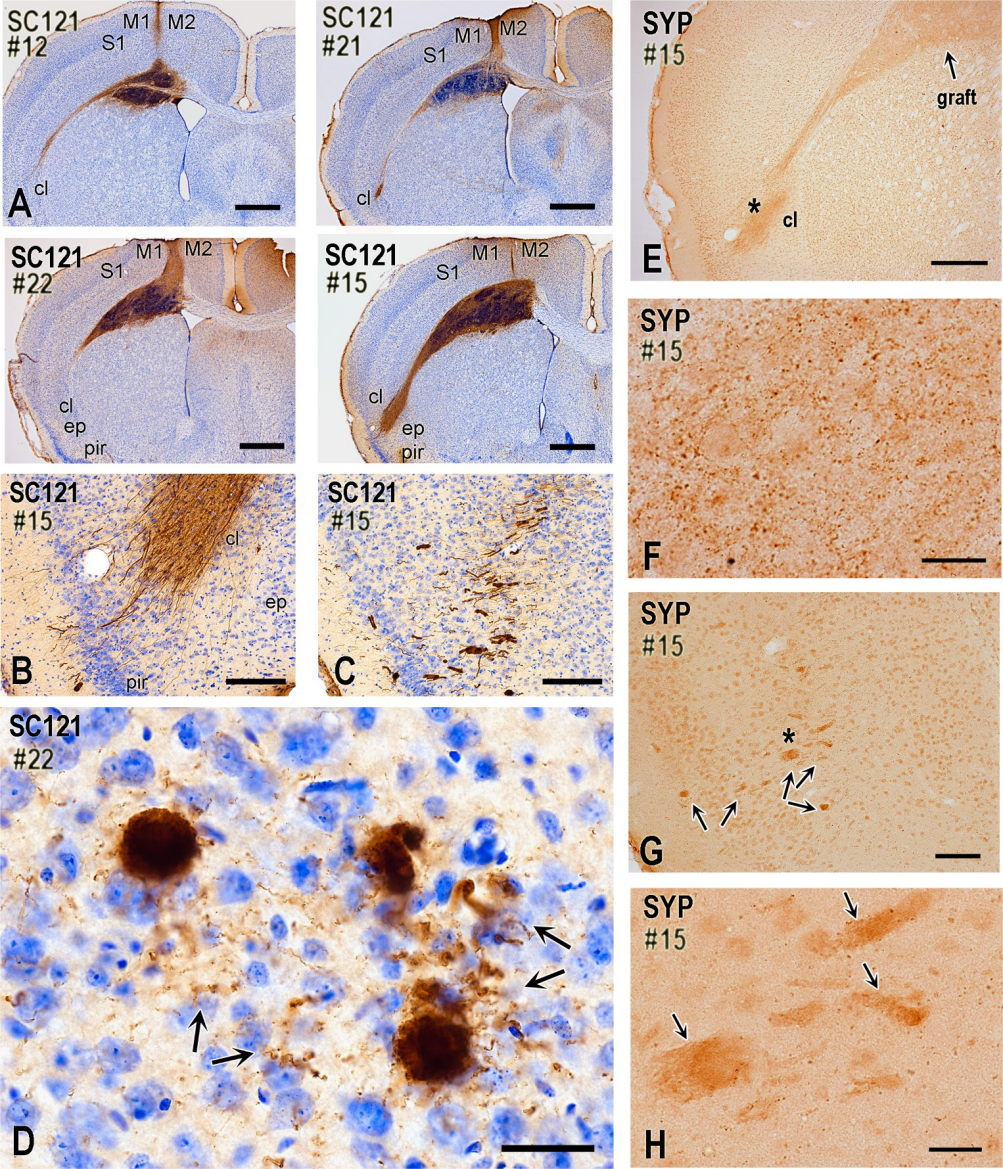
Neuronal differentiation of ChR2-transduced and non-transduced hNP transplants and pathfinding of axons from their neuronal progenies two months after transplantation (mice). (A) These four panels are from representative cases visualized with IHC for the human-cell specific epitope SC121 and illustrate a consistent engraftment of hNPs in the primary motor area at a coronal level corresponding to caudal septum. Transplant engages deep cortical layers, medial corpus callosum, and dorsal neostriatum. In most cases, the transplantation needle track is also evident. Panels also show that SC121^+^ transplant-derived axons project along the fibers of corpus callosum and exit at the level of claustrum. Transplants in #21 and #22 contain transduced hNPs, whereas in #12 and #15 contain non-transduced cells. All cases are from injured mice. (B-D) SC121 immunohistochemical preparations illustrating the propensity of transplant-derived axons to exit the lateral corpus callosum at the level of the claustrum and then course in deep insular/piriform cortex. Case 15 involves non-transduced NPs. There is a striking concentration of axons at the claustrum, and an occasional further advancement towards the insular and piriform cortex. In some cases, bundles of axons extend all the way to layer II of piriform/insular cortex (B), commonly following a course oblique or vertical to the plane of view (C). Panel D shows that axon bundles branch into numerous single axons with boutons (arrows) in the deep insular/piriform cortex (Case 22, transduced NPs). (E-H) These human synaptophysin-immunostained preparations show the formation of early synaptic fields by transplant-derived neurons. Low-level synaptophysin expression often extends from the site of the transplant all the way to the lateral corpus callosum and insular/piriform area, with an apparent terminal field at the claustrum/piriform cortex (E). With higher magnification, terminal field contains putative synaptic boutons (F; photograph is taken from the claustral/piriform area indicated with asterisk on panel E). Bundles of axons that course obliquely or vertically to the coronal plane as in panels C-D also express diffuse synaptophysin immunoreactivity (G; bundles are indicated with arrows). Synaptophysin^+^ boutons surround such bundles (H; panel is a magnification of area indicated with an asterisk on panel G). M1, primary motor cortex M2, secondary motor cortex; S1, primary somatosensory cortex; cl, claustrum; ep, endopiriform nucleus; pir, piriform cortex; SYP, synaptophysin. Scale bars: A, 800μm; B-C, 200μm; D, 30μm; E, 400μm; F-H, 20μm; G, 100μm.

In mice, graft survival was excellent (Figs 8 and 9). Viral hChR2 transduction and the presence or absence of IA injury had no apparent impact on the fate of transplanted hNPs. Twenty % of hNPs at the transplantation site were positive for the mitotic marker Ki67, indication of an ongoing proliferative potential of the transplant; all such cells were located in neuroepithelial rosettes that were still visible at the transplantation sites. On dual fluorescence preparations for human markers and markers of neural differentiation we found that 63% (44–96%) of SC121^+^ cells and 66% (57–72%) of HuNu^+^ cells expressed TUJ1, a type III β-tubulin epitope that marks neurons from an early stage **(Fig 8A)**. There was some penetration of hNP-derived cells with neuronal features in the halo around the transplants, but no evidence of long-distance migration of SC121^+^ (transplant-derived) cells. Even when hNPs were inadvertently engrafted in the ventricle or leaked over pia, neuronal differentiation rate was high at 57% (45–74%). Less than 5% of transplanted cells were immunoreactive for the astrocytic marker GFAP, the early oligodendrocytic marker PDGFRα or the mesenchymal marker BMP4. In transplants of transduced hNPs (hChR2-hNPs), we found that 62% (60–90%) of transplant-derived neuronal cells expressed hChR2-YFP signal **(Fig 8B)**. In areas of dense human synaptophysin (hSYP)^+^ boutons we occasionally found GABA immunoreactivity manifesting as punctate perikaryal and perhaps also neuropil staining; transplants were not immunoreactive for vGLUT1 (data not shown).

Transplants from hChR2-hNP injections formed in cortical layers 5/6 and the adjacent corpus callosum **(Fig 9A)**. Neuronal cells differentiated from transplanted hNPs extended axons primarily along the corpus callosum, mainly on the ipsilateral but to some extent also the contralateral side **(Fig 9A)**. Axons were also seen in the cingulum, internal capsule and fornix. Invariably, callosal axons exited the external capsule at the dorsal claustrum and then bundled in cylindrical structures that appeared to course in the mediolateral and then caudally in the anteroposterior axis; some of these tubes reached piriform cortex **(Fig 9B–9C)**. These structures have a complex 3D configuration and branch off to individual axons with bouton-like synaptic structures **(Fig 9D)**. Human synaptophysin IHC confirms the synaptic maturation potential of the hNP transplants and reveals terminal fields in claustrum **(Fig 9E and 9F)** and in deep insular/piriform cortex **(Fig 9G and 9H)**. In some cases we also saw terminal fields in nearby neocortical and cingulate areas.

**Fig 10:**
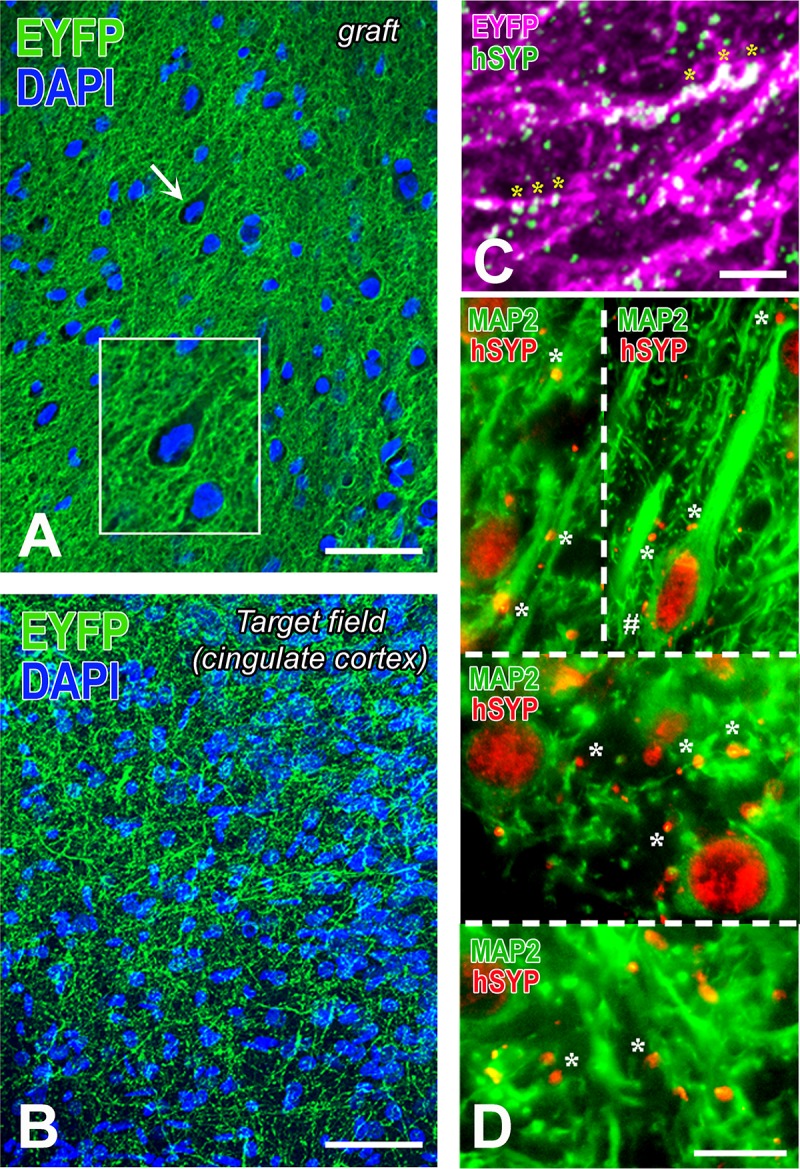
Differentiation of hChR2-hNP transplants in rat motor cortex and patterns of innervation of host neurons. (A-B) These YFP-immunostained preparations show the distinct features of hChR2^+^ neural tissue in the transplant (A) versus a hChR2^+^ terminal field in cingulate cortex (B). YFP expression in (A) is restricted to the membrane of the neurons and their processes. The latter form a dense neuropil with webs of processes filling the space between neuronal cell bodies. Some of these hChR2-hNP-derived neurons, for example the one indicated with the arrow and enlarged in the inset have cortical features. The terminal field of hChR2-hNP-derived neurons in cingulate cortex (B) has a very different appearance than the “neuropil” in the transplant and it obeys the cytoarchitecture of host cortex. No perikaryal-type staining is encountered, evidence that graft-derived neurons have not migrated into host cortex. (C) This dually stained preparation for two transplant-selective neuronal markers (YFP immunoreactivity for hChR2 and human synaptophysin immunoreactivity) is from layer II of host cingulate cortex and demonstrates both the dense terminal field and the extensive colocalization of the two markers in transplant-derived axons and their processes (asterisks; double labeling is white here). A larger panel showing more of this terminal field is in S6 Fig. (D) These dually stained preparations from host motor and cingulate cortex with MAP2 for neurons and human synaptophysin for transplant-derived terminals show a very large number of terminals apposing dendrites and dendritic branches of host neurons in layer 2–3 of motor cortex (top left), layer 5 of motor cortex (top right), deep layer 2–3 of cingulate cortex (middle) and superficial layer 2–3 of cingulate cortex (bottom). In many cases, dendrites are sectioned transversely. There are occasional terminals on the perikarya of large neurons (see an example on a pyramidal neuron labeled with #). Scale bars: A-B, 50μm; C, 10μm; D, 20μm.

**Fig 11:**
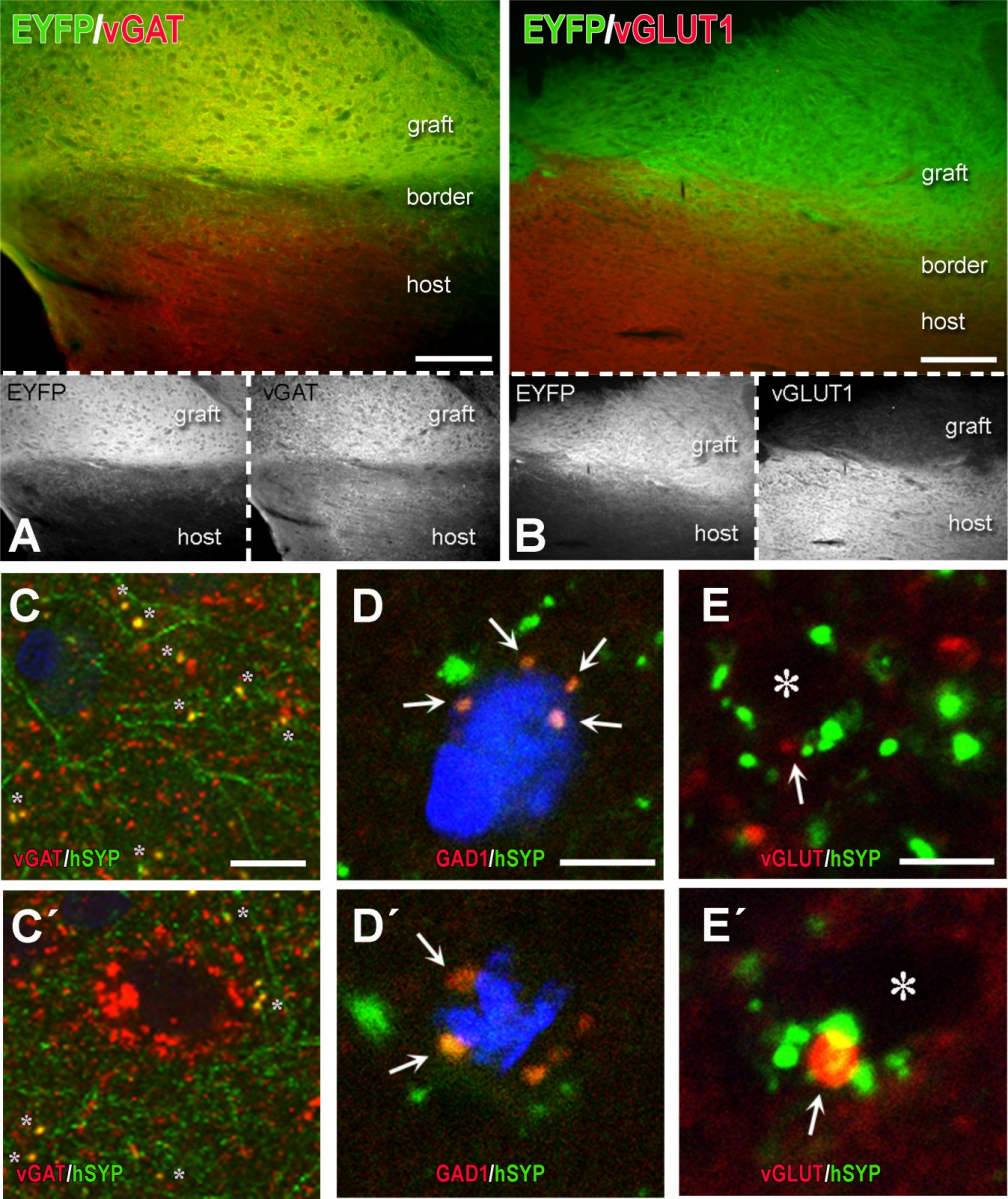
Partial characterization of neurotransmitter identity of differentiated hChR2-hNP transplants and their terminals in host brain. (A-B) These are dually immunostained preparations for YFP (hChR2 marker to label the transplant and transplant-derived structures) and either vGAT, a presynaptic marker of GABAergic neurotransmission (A) or vGLUT1, a presynaptic marker of glutamatergic neurotransmission (B). Color panels show both transplant and neurotransmission marker immunoreactivities, whereas black and white panels underneath the color ones illustrate transplant and neurotransmitter markers separately. Note the dense GABAergic neurotransmission in the graft (orange neuropil in A) as contrasted with the very low glutamate marker immunoreactivity (B). (C-E´) Representative images illustrating the GABAergic or glutamatergic differentiation of transplant-derived human synaptophysin^+^ (hSYP^+^) terminals in rat motor cortex. Differentiation was assessed with IHC for GABAergic markers such as vGAT (C-C´) or GAD1 (D-D´) and glutamatergic markers such as a mixture of antibodies for vGLUT 1 and 2 (vGLUT) (E-E´). Images in C-C´ are taken at a lower magnification than D-E´. There are multiple vGAT^+^ or GAD1^+^ transplant-derived hSYP^+^ terminals (yellow color), most of them on non-identifiable host structures, probably dendrites (asterisks in C-C´) but quite a few also on somata (double asterisk on C´ as part of a basket-type GABAergic innervation of a rat cortical neuron; arrows on D-D´). At the contrary, we were not able to find any colocalization of vGLUT in hSYP^+^ terminals (D-D´); even when there is double labeling as in E´, the relationship is that of juxtaposition, not colocalization. Asterisks on E-E´ are unlabeled rat (host) structures, probably dendrites. Scale bars: A-B, 100μm; C-E, 10μm.

**Fig 12:**
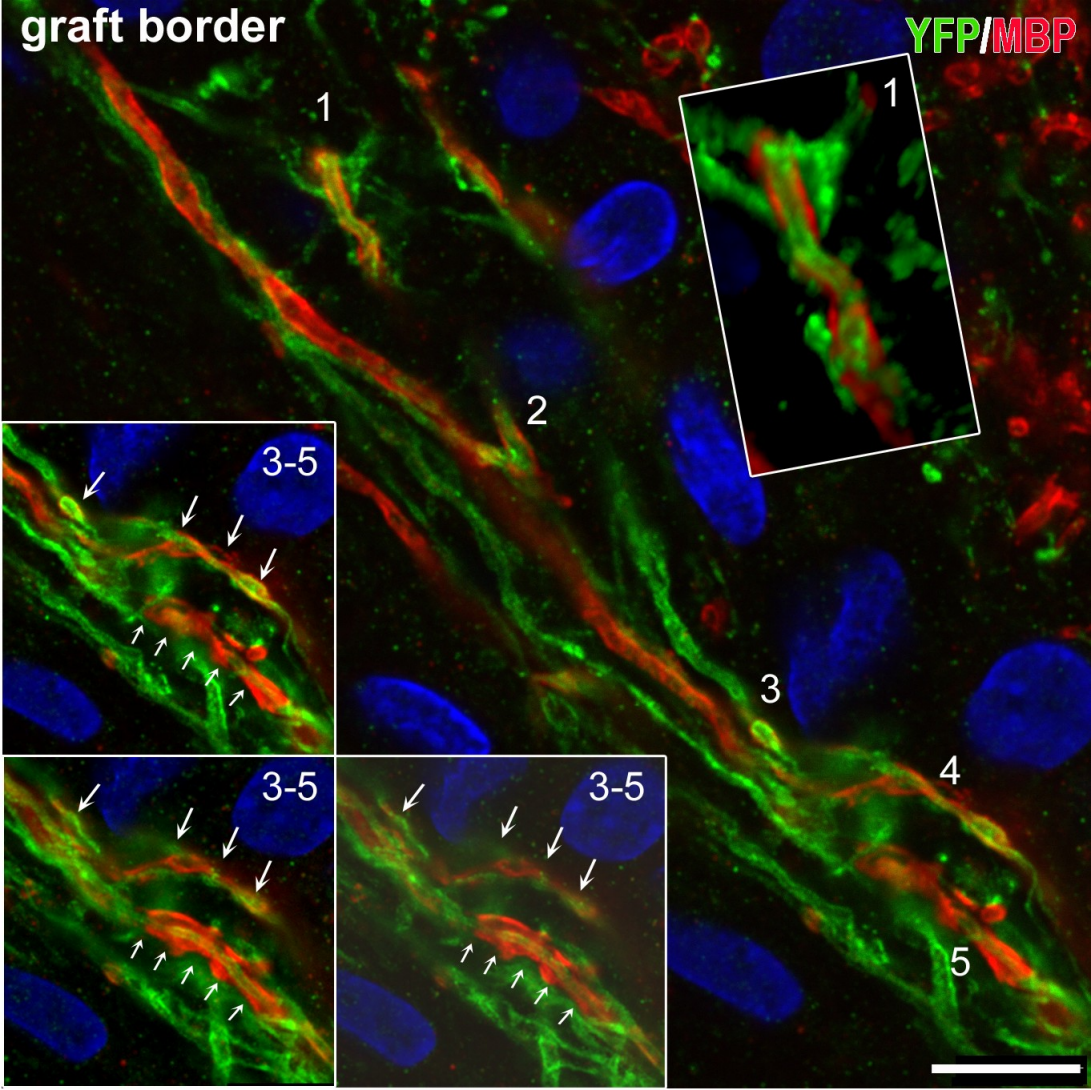
Myelinated human axons in dually immunostained preparations with YFP antibodies (for hChR2^+^ axons) MBP (for myelin) through the border of a transplant. Single optical sections are taken with a confocal microscope. Human ChR2-hNP-derived axons are in green and myelin in red. At least a third of these axons is myelinated. Inset on top right is a 3D rendition of profile 1 in main frame from z-stack. Insets on bottom left are optical sections of profiles 3–5. Note the non-continuous myelination in the form of serial MBP^+^ “cuffs”. Graft is on the left. Rat corpus callosum is on top right. Scale bar: 10μm.

**Fig 13:**
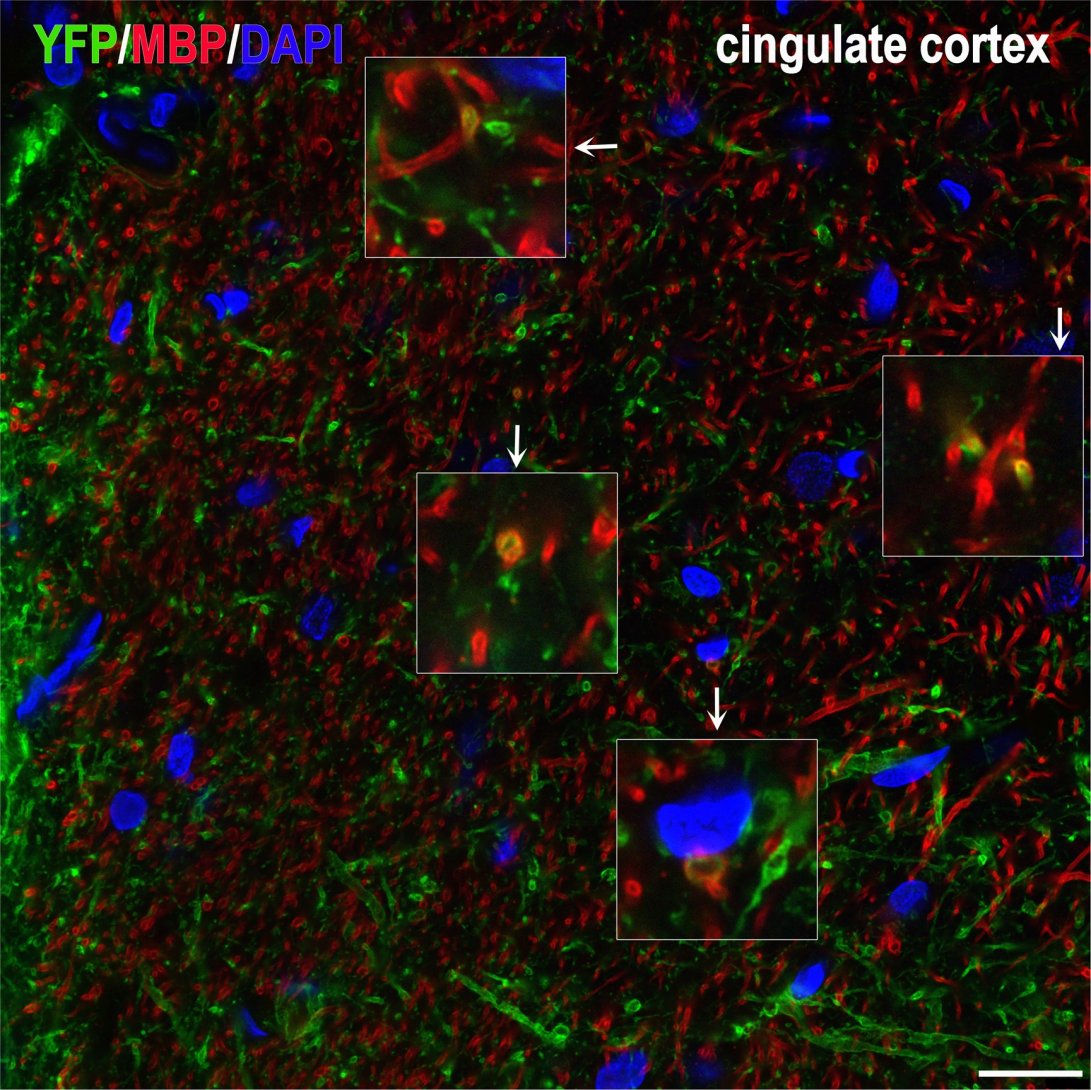
Myelinated human axons deeply into host cortex. This section was immunostained and imaged exactly as section in **Fig 12**. Here we illustrate a terminal field of transplant-derived neurons in cingulate cortex. Note the occasional double-labeled profiles, suggesting a low degree of myelination of transplant-derived axons away from the graft. Insets are magnifications of profiles in main frame linked with arrows. Scale bar: 20μm.

**Fig 14:**
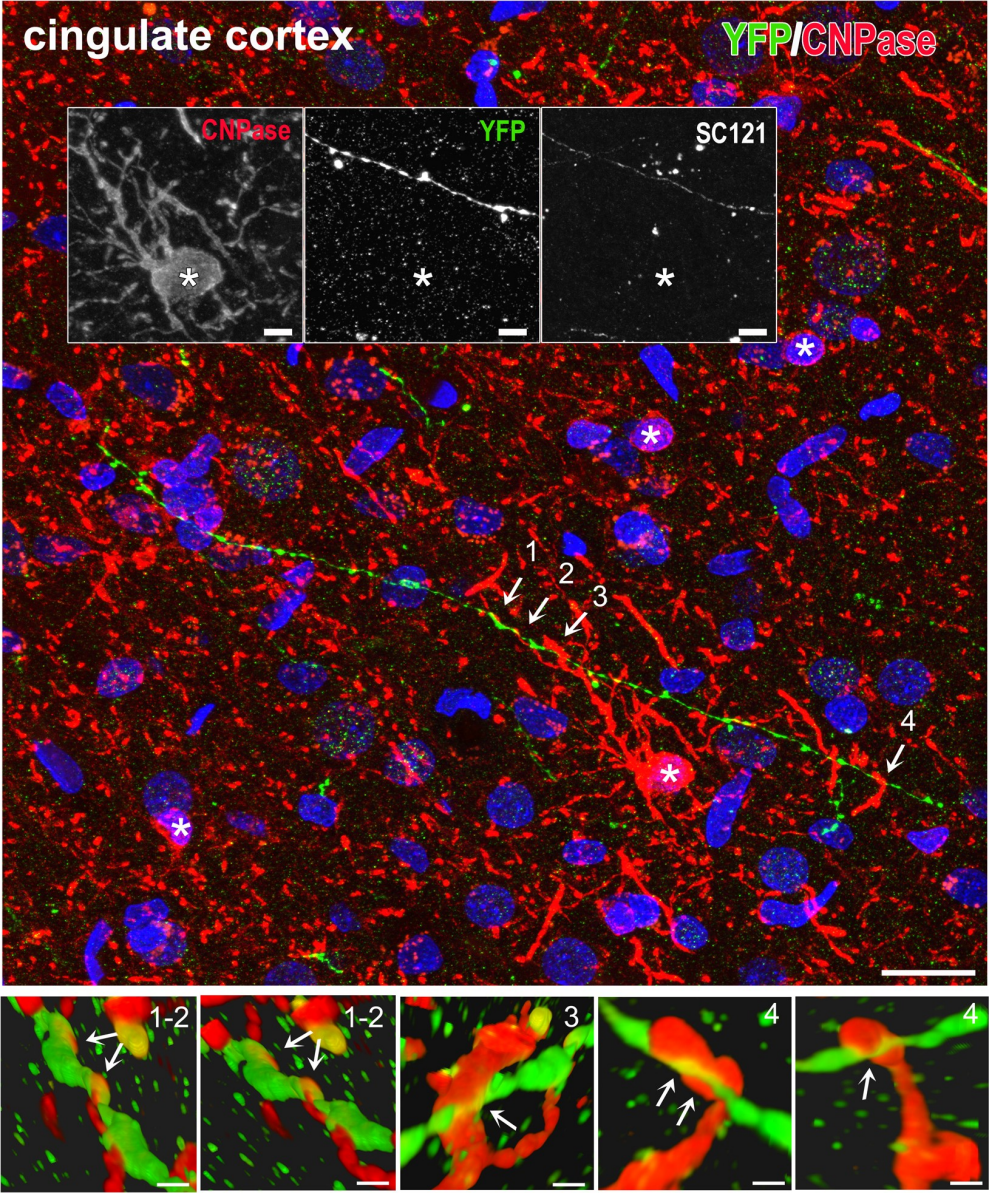
An example of ensheathing-type contacts made between a transplant-derived axon and several host-derived (rat) oligodendrocytic processes in host cingulate cortex. Maximum projection confocal image was captured from a section triply immunostained for YFP for transplant-derived axons (green), CNPase for oligodendrocytes (red) and SC121 for transplant-derived cells (white). (Top panel). Although four oligodendrocyte cell bodies are identified (asterisks), it appears that many of the contacts are made by the centrally located cell. None of the red oligodendrocyte profiles was double labeled with SC121, indication that they were of host origin (the lack of SC121 immunostaining of the central profile is shown in inset, against the SC121 immunoreactivity of the human axon). For the sake of conveying a sharper color, white was omitted from the panel. Examples of ensheathing contacts are indicated with numbers 1–4. (Bottom panels). Here we performed 3D reconstructions of ensheathing profiles 1–4 to show the detailed configuration of the human axon and the host oligodendrocytic processes enveloping it. Ensheathing profile 3 has unusual morphology, but this may be related to the immaturity of the process. Scale bars: Top panel, 20 μm; insets, 5μm; bottom panels, 1μm.

In rats, because of the longer survival times, emphasis was on terminal fields and neurotransmitter differentiation. Brain tissues were analyzed with IHC for: the localization and differentiation of grafted cells (HuNu, SC121, or TUJ1 immunoreactivity); expression of hChR2 (YFP immunoreactivity); and the presence and distribution of human synapses (human-specific synaptophysin immunoreactivity). In all cases, we found an extensive differentiation of transplanted cells into neurons (**Fig 10A** and **S4 Fig**). We rarely saw SC121^+^ or hChR2^+^ neurons in the halo around the border of the graft and we saw no such neurons in remote sites, a pattern indicating lack of migration. Human NP-derived neurons inside the transplant projected axons outside its boundaries and established dense terminal fields of hChR2+ axons and terminals in cingulate and motor cortices (**Fig 10B**), whereas many axons entered the corpus callosum. Two independent markers of transplant-derived axons and terminals, i.e. human ChR2 and hSYP expression, colocalized extensively in host terminal fields; human ChR2 (marked by YFP immunoreactivity) was preferentially expressed in axons and axon bundles, whereas human synaptophysin was abundant in axons but also terminal boutons attached to YFP^+^ structures (**Fig 10C** and **S5 Fig**). The majority of transplant-derived synapses were in contact with host dendritic profiles (**Fig 10D**). In an initial characterization of the neurotransmitter phenotype of the differentiated transplant, we found intense vGAT and very low vGLUT1 immunoreactivity in the transplant, evidence for a predominantly GABAergic differentiation of the graft (**Fig 11A and 11B** and **S6 Fig**). Many transplant-derived terminals in the host (in motor and cingulate cortex) were also positive for GABAergic markers such as vGAT (**Fig 11C and 11C´** and **S7 Fig**) and GAD1 (**Fig 11D and 11D´**) whereas we found no colocalization of human synaptophysin with glutamatergic markers, i.e. vGLUT1/2 (**Fig 11E and 11E´**) with high-resolution confocal microscopy. Some large axons in the vicinity of the graft (**Fig 12** and **S8 Fig**) and smaller axons in terminal fields in the motor and cingulate cortices **(Fig 13)** were myelinated, but most graft-derived axons were unmyelinated. Oligodendrocytes differentiating from the hNP graft did not migrate far away from the transplant site (**S9 Fig**), suggesting that the myelination of transplant-derived axons in terminal fields could not be attributed to these fellow oligodendrocytes. Indeed, in host cortex, we found SC121^-^ oligodendrocytes contacting human axons with classical ensheathing configurations (**Fig 14**). Inside transplants or at their border, double labeling with MBP and SC121 occasionally showed punctate SC121 immunostaining in myelin sheaths, although most sheaths were SC121 negative. At the transplant border, occasional SC121^+^ oligodendrocytes were seen to contact transplant-derived axons, although some contacted axons were SC121 negative (**S10 Fig**).

### Optical stimulation experiments: responses of hChR2-hNP-derived neurons to light

Before starting optogenetic stimulation experiments with hChR2^+^ neurons, we first ensured that CHR2 expression did not affect their neuronal properties. We found that membrane input resistance, AP firing rate, AP threshold and AP half width, were not statistically different between cells with and without CHR2 expression **(S3C and S3D Fig)**, suggesting that ChR2 expression does not affect the electrical properties of hNP-derived neurons.

**Fig 15:**
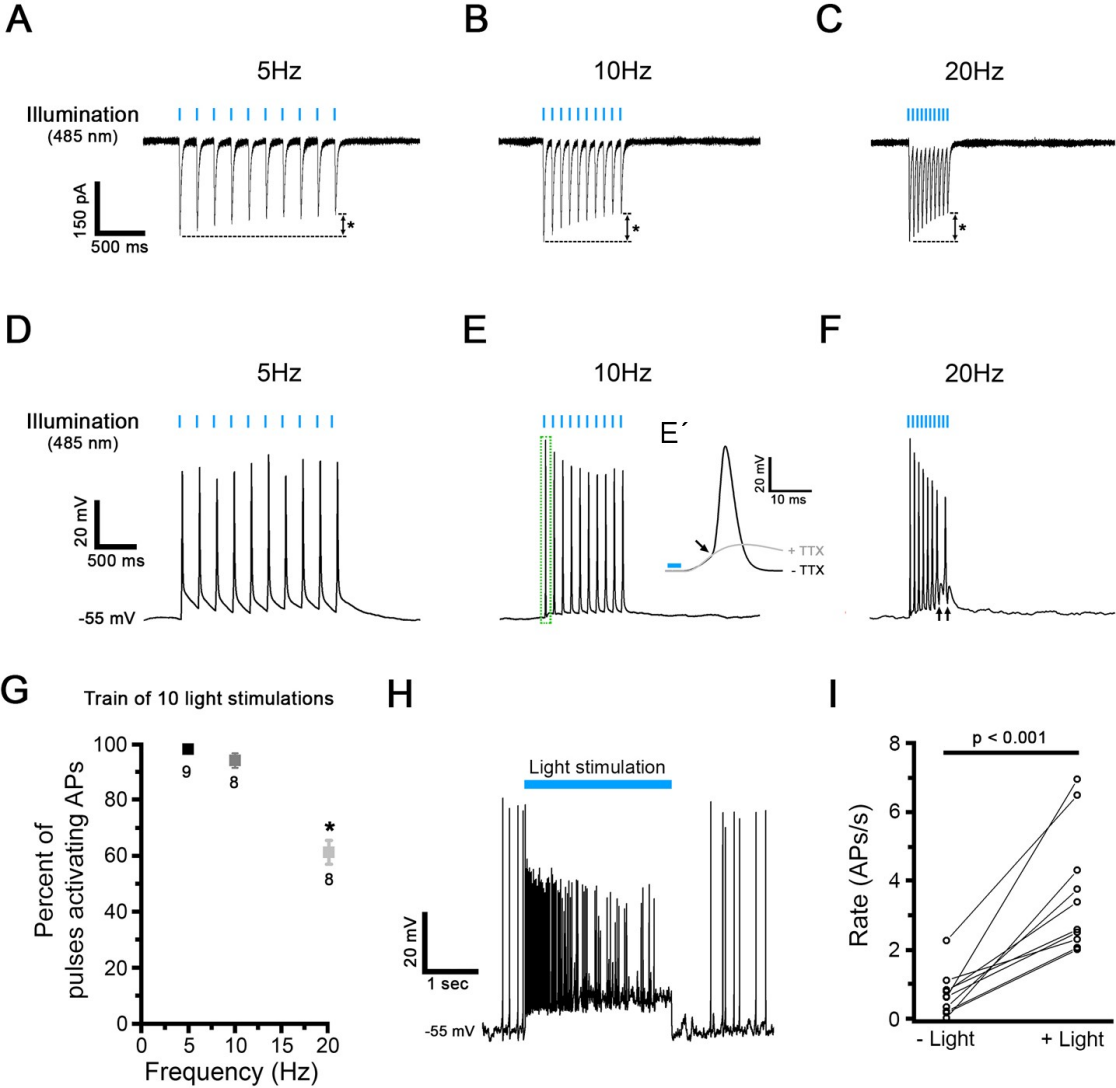
Electrophysiological recordings and optogenetic light stimulation of hChR2-hNP-derived neurons differentiated *in vitro*. (A-C) Representative voltage clamp traces in response to blue light stimulation (485 nm) at 5 Hz (A), 10 Hz (B) and 20 Hz (C) obtained in hChR2-hNP-derived neurons. The amplitude of the 1^st^ pulse response was similar at each tested stimulation rate (for example, 269.8 ± 42.2 pA at 5 Hz). However, response amplitudes decreased during the 10-pulse stimulation, by a total of 29–36% (from 1^st^ to 10^th^ pulse), with an increasing level of adaptation at higher stimulation rates (asterisks). (D-F) Representative current clamp traces in response to a train of light stimulation at 5 Hz (D), 10 Hz (E) and 20 Hz (F). Before light exposure, the membrane potential was preset to -55 mV by current injection. When TTX is added to the extracellular solution AP generation is blocked (E´). (G) Comparison of spiking fidelity in response to different rates of 10-pulse optogenetic stimulations. (H) During a single 2.5s-long light stimulation, the AP firing rate initially increases and then adapts. (I) During a 2.5 s-long single light stimulation (+light), AP firing rate significantly increased from 0.7 ± 0.2/s (-light) to 3.6 ± 0.6/s (n = 10; p < 0.001, paired t-test).

For optical stimulation, hChR2-expressing neurons were exposed to 10-pulse stimulations with 485 nm light, 5 ms each, at different pulse rates. Ten-pulse protocols were applied 5 times, every 4 seconds, and responses were averaged for analysis. In voltage clamp, such pulse trains activated inward currents at every individual stimulus, however, the inward current amplitude decreased during the 10-pulse stimulation, more prominently at higher stimulation rates **(Fig 15A–15C)**. In voltage clamp, light-activated inward currents were not affected by TTX (n = 3, data not shown), assuring that these currents were caused by ChR stimulation. In current clamp, pulse trains activated TTX-sensitive APs **(Fig 15D–15F).** The AP amplitude decreased during 10-pulse stimulation, more prominently at higher stimulation rates. Additionally, at higher stimulation rates, the success rate of AP activation was reduced (**Fig 15F–15G**), as indicated by arrows in **Fig 15F**. To test the efficiency of a single long pulse compared to pulsed stimulation, a 2.5 s long light pulse was applied **(Fig 15H and 15I)**. Compared to spontaneous firing before optical stimulation, single pulse stimulation increased AP firing rates significantly, about 5-fold **(Fig 15I)**. However, during stimulation, AP amplitudes decreased to ~ 60%, and firing rates adapted.

These data show that optogenetic train stimulation efficiently induces AP firing in hChR2^+^ neurons, and that train stimulation at higher rates and with longer pulses may become less effective.

### Optical stimulation experiments: light activation of hChR2-hNP-derived neurons drive postsynaptic activity *in vitro*

**Fig 16:**
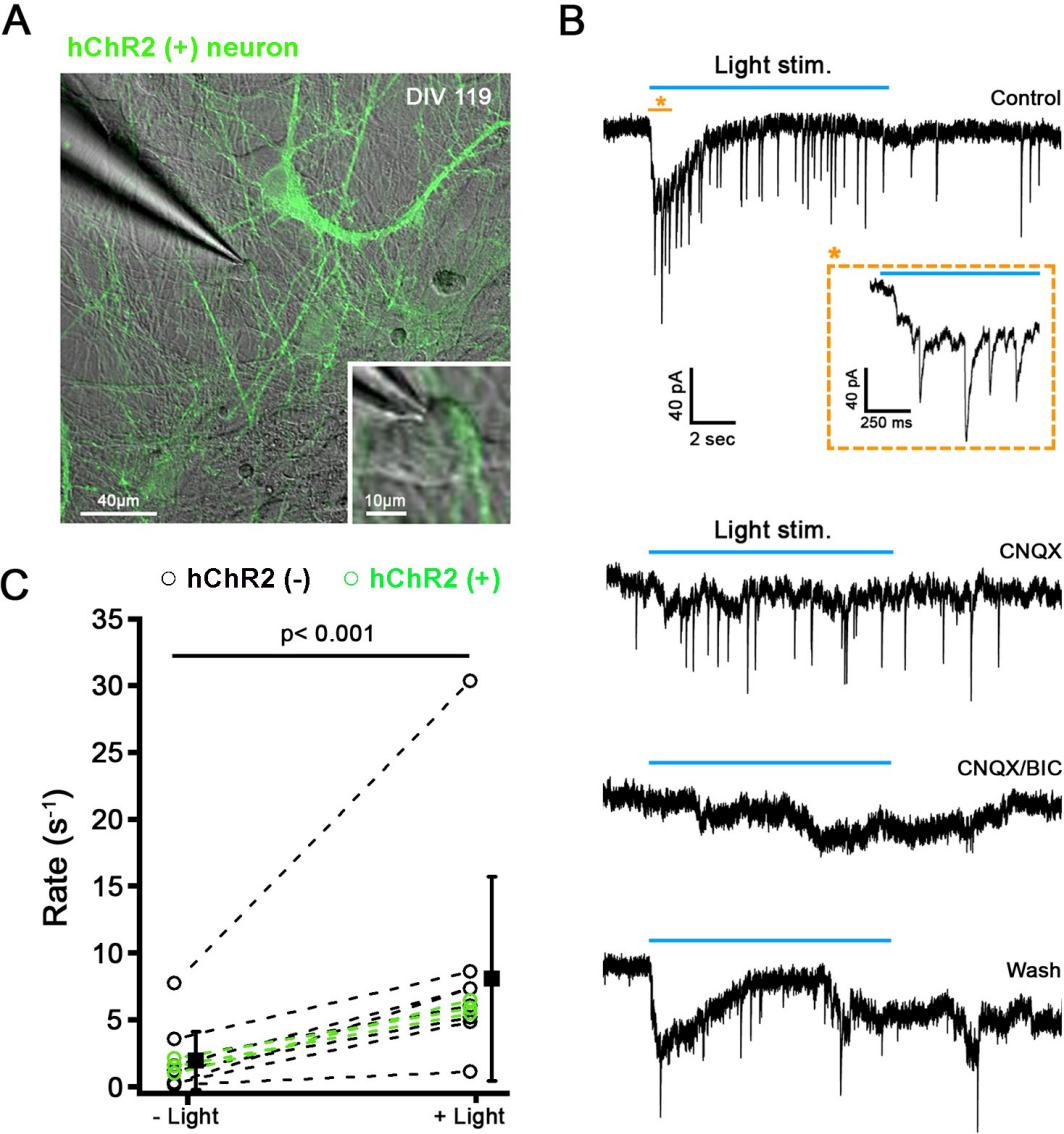
Light stimulation of hChR2-hNP-derived neurons triggers postsynaptic events in ChR2^-^ and ChR2^+^ neurons. (A) Confocal image showing ChR2^+^ neurons (green) in co-culture with ChR2^-^ neurons after 119 days *in vitro*. (B) Example of a voltage clamp recording from a ChR2^-^ neurons in co-culture with ChR2^+^ neurons; same cell as shown to be recorded in A. In response to light stimulation (blue bar), a slow inward current and synaptic events were triggered in control conditions (top trace). Inset shows an extended time scale of the recording time. The postsynaptic responses were blocked by the presence of CNQX and bicuculine suggesting that they were due to light stimulation of presynaptic hChR2^+^ neurons. (C) Synaptic rate before and during light stimulation plotted for individual recordings of ChR2^-^ neurons (n = 8) and ChR2^+^ neurons (n = 3). Solid black squares represent mean of combined ChR2^-^ and ChR2^+^ neurons, respectively (mean ± SD). For ChR2^-^ neurons, the synaptic rate increased about 4-fold during light stimulation, from 2.2 ± 2.4 to 8.9 ± 8.4 events/s (n = 8; p < 0.05, paired t-test).

Next, we investigated whether light activation of hChR2^+^ neurons can drive postsynaptic activity in co-cultured hChR2^-^ neurons. Human ChR2^+^ neurons were stimulated with a 10s blue light pulse while recording from a co-cultured hChR2^-^ neurons **(Fig 16A and 16B)**. Fourteen % of recorded cells (8/56) displayed an increased rate of postsynaptic events in response to light stimulation **(Fig 16B and 16C)**. From a spontaneous rate of 2.2 ± 2.4 events per second, during light stimulation, the rate of postsynaptic events increased about 4-fold, to 8.9 ± 8.4 events per second (n = 8; p < 0.05, paired t-test) (**Fig 16C**, black lines). The combined application of CNQX and Bicuculline reversibly blocked both spontaneous and light stimulated synaptic activity **(Fig 16B),** suggesting that the light stimulated events were generated by light stimulation of the presynaptic hChR2^+^ neurons. Additionally, recordings with light stimulation were also performed directly from hChR2^+^ neurons. As expected, these cells responded with an inward ChR2 current directly to the light stimulation, as shown in the previous figure **(Fig 15A)**. In 18% of the hChR2^+^ neurons (3/16), from a spontaneous rate of 1.6 ± 0.6 events/s, during light stimulation, the rate of postsynaptic events increased about 4-fold, to 6.0 ± 0.5 events/s (n = 3) **(Fig 16C, green lines)**. Again, postsynaptic events were abolished by application of CNQX/Bicuculline (data not shown).

*In vivo* stimulation of transplants in rats was inconclusive. When we stimulated these transplants with light pulses of 20 Hz for 15 minutes using a custom LED device 1 mm distance from the surface of the brain right above the exposed transplantation site and analyzed tissues through the level of the transplant for c-FOS immunoreactivity one hour later, results were mixed. The expression of c-FOS in transplants was variable and did not allow the detection of an obvious effect of light stimulation in the host. For example, we saw non-stimulated transplants with high c-FOS immunoreactivity and stimulated transplants with no c-FOS immunoreactivity (**S11–S13 Figs**). Upon closer look we found that, in stimulated transplants, immunoreactive nuclei were uncommon in areas with dense synaptic activity (**S14 Fig**), a finding consistent with local inhibitory neurotransmission.

Overall, these results suggest that optical stimulation of hChR2^+^ neurons *in vitro* can evoke postsynaptic events in co-cultured hChR2^-^ and hChR2^+^ neurons via established functional synapses.

## Discussion

The present study demonstrates the successful generation of optogenetically competent hNPs suitable for transplantation, the stable expression of the optogene down to their neuronal progenies, and the responsivity of such progenies to light. With the method we employed here, hNPs were transduced at an advanced stage of differentiation, a strategy ensuring sustained transduction through the course of the neural lineage and the future expression of hChR2 and reporter (YFP) transgenes under synapsin promoter. These optogenetically competent hNPs were fully differentiable into mature, light-responsive neurons, with both excitatory and inhibitory phenotypes in vitro but evolved predominantly into inhibitory GABAergic profiles after grafting *in vivo*, where they established dense terminal fields. Human ChR2-hNP derived neurons were able to generate postsynaptic responses *in vitro*, although demonstrating such effects *in vivo* is technically challenging and would require additional efforts.

Since optogenetics have been introduced as a methodology in the neurosciences in 2005 [38], a variety of neural cells have been transduced by lentivirus or AAV and then transplanted into animal models for the purpose of exploring connectivity and function or correcting disease-related phenotypes. Examples include ES- and IPS-cell-derived NSCs, NPs or neurons from human [16, 39–46] or mouse [47, 48], and neurons directly converted from fibroblasts [49]. In the majority of these studies, outcomes of interest (especially physiological) were assessed on *ex vivo* slices [39, 41–46, 48, 49], although a few studies attempted to examine select structural and functional outcomes *in vivo* [16, 39–41, 47]. These *in vivo* studies involved transplantation in models of disease including temporal lobe epilepsy (TLE), peripheral paralysis and stroke [16, 41, 47], but some work explored the formation of connections [40]. Compared to our experiments here, Byers and colleagues used iPSCs for ChR2-YFP transduction and FACS-sorted ChR2-YFP-iPSCs were differentiated to NSCs which they allowed to differentiate for close to a year after graft in the striatum of female nude rats [40]. Although only 15% of grafted ChR2^+^ NPs differentiated into neurons and terminal fields were sparse in their hands, they found fMRI responses to light stimulation both locally (striatum) and distally (apparently not consistent with the known projections of the rat striatum) [40]. These problems may suggest epigenetic modification and silencing of ChR2-YFP expression in the process of differentiation from ES cells to NPs. Altogether, testing optogenetic efficiency *in vivo* has been extremely difficult and our unsuccessful *in vivo* efforts summarized four paragraphs below bear testament to the difficulty of the task.

One of the challenges in this study was our difficulty in generating stable, optogenetically competent hNP cell lines by cloning at the hES stage because of problems with gene expression. This problem is consistent with the experience of Weick and colleagues who found that the expression of ChR2 transgene in their ChR2-hESC line was low and ChR2-mediated currents were relatively small at 12 weeks *in vitro* compared to transiently transduced NPs [45]. In further communication with the authors, we learned that ChR2 expression using this line was unreliable, possibly because of dilution of expression during expansion of hES cells and epigenetic modification during neural differentiation (Zhang SC, personal communication). The paucity of studies where investigators employed clonal optogenetically competent cell lines [40, 44, 45] and apparent challenges with differentiation and integration after transplantation may be an indication of problems with stable gene expression over successive generations of cells. Here, these challenges were obviated by employing transduction at a later stage (hNP stage). In the future, this problem may be resolved by knock-in gene editing of ES cells by CRISPR to target the optogene to specific DNA location and minimize gene silencing. Another issue is the determination of the hNP stage at which transplantation will reliably result in the desired neuronal outcome. In the present study this was not such a big problem but, as we have indicated in our previous work [5], non-clonal hNPs are best monitored with immunophenotyping by flow cytometry in order to establish a stage in culture that maximizes differentiation efficiency *in vivo*. For example, relatively late hNPs with TnTx^+^nestin^+^ phenotype, but not earlier ones with CD15^+^TnTx^-^ signatures, are more likely to generate predominantly neuronal transplants [5].

Human NPs generated in this paper were cued into a forebrain fate choice because of the anticipated transplantation into cortical sites. Regardless of the genuine “forebrain” signature of thus generated neurons, hChR2-hNP-generated nerve cells express both excitatory and inhibitory neurotransmitter markers that render them extremely useful as tools to ascertain the functionality of *in vivo* generated circuits after transplantation. Our *in vitro* experiments show that hChR2^+^ neurons can be activated by light but still display some immature features, as indicated by their depolarized resting membrane potential. This can be partially explained by the fact that electrophysiological recordings have been performed at room temperature, where kinetics and conductances of ion channels are reduced compared to body temperature, rendering neurons less excitable [50, 51]. This condition may explain why the success rate of AP activation is significantly reduced during light train stimulation above 10 Hz. Our *in vitro* experiments also show that light stimulation of hChR2-hNP-derived neurons can generate synaptic activity in neighboring non-ChR2 hNP cells, providing evidence that hNP-derived neurons establish functional synaptic connections with other neurons in culture. Upon light stimulation, we found both glutamatergic and GABAergic postsynaptic activity. These results are consistent with previous ex vivo work on slices prepared from brains transplanted with ChR2-engineered hNPs showing the generation of both excitatory and inhibitory post-synaptic currents in ChR2^-^ neurons after light stimulation [45, 52, 53].

Our *in vivo* findings in mice indicate a prodigious differentiation of hChR2-hNPs into neuronal cells, with less of a presence of astrocytes, oligodendrocytes or other cells, at least at 2–4 months post-transplantation. At this time point, there are still mitotic neuroepithelial profiles at the transplantation site and about a fifth of cells still undergo mitotic divisions. Indeed, it appears that most hChR2-hNP transplant-derived cells that do not express neuronal markers are dividing neuroepithelial cells. It is possible that such dividing progenitors may give rise to non-neuronal cells at later time points and it is highly likely that they are plastic enough to generate non-neuronal cells, e.g. astrocytes, if injected into white matter, as we have seen previously. Continued mitotic activity and ongoing expansion of the transplant was also observed in our previous work in the brain and spinal cord [5, 10] at 3 months, but subsided at 6 months. Based on this earlier work, early neuronal differentiation to a TUJ1^+^ phenotype was achieved by 3 months [5, 10], a pattern consistent with the high rates of neuronal profiles encountered in the present study. Although pathfinding of newly generated axons along callosal trajectories has been observed in the case of hNP transplants in cortex [36, 54], a very interesting finding in our experiments in mice featured by deeper cortical grafts is the consistent exit of axons tracking in the corpus callosum at the level of the external capsule into the territory of claustrum and the endopiriform area when the hNPs were transplanted in and around the corpus callosum. Such axons seem to establish synaptic fields in the claustrum, but they continue to pathfind and distribute terminals, seemingly lateral- or caudal-ward in piriform and insular regions. The presence or absence of the ChR2 transgene or experimental history (naïve versus TBI) did not appear to play significant roles in the pathfinding profile of transplanted cells. In concert, preparing hNPs in the way described here is very effective in biasing these cells to early neuronal fates with pathfinding and synapse-forming capabilities within cortical-limbic circuitries, though it is possible that remaining populations of dividing neuroepithelial cells may give rise to non-neuronal cells at later time points.

In rats, perhaps due to the longer time of graft maturation (9 instead of 2–4 months), transplants were generally more differentiated, with very few neuroepithelial formations. Using smaller numbers of cells typically helps with tumorigenicity in long-term survival animals according to our previous investigation [5] but here we opted for higher numbers because it was the first time with this cell preparation and our primary goal was to ensure survival. Neuronal differentiation was remarkable. However, there were places with adipocytic differentiation more superficially, where grafting was in close proximity to the pia, and there were patches of astrocytic and some oligodendrocytic differentiation associated with the neuronal components of the transplants. Terminal fields were established primarily in the adjacent motor and cingulate cortices, a pattern that is different than grafts in mice and may correspond to the more superficial location of the rat grafts away from the corpus callosum. This is most probably a site-specific rather than a species-specific diversion, since grafts injected deeply in rat neocortex are known to also enter the corpus callosum and form terminal fields in the claustrum and adjacent areas [40]. We also found that some large axons near the graft and smaller axons at the terminal fields were myelinated, in part by host oligodendrocytes. The latter serves as yet another indication of integration of transplant-derived axons with host tissue (rat neocortex). Although some graft-derived Olig2^+^ oligodendrocytes were seen to contact both human and some host axons at the border of the graft, this was a rather rare phenomenon, perhaps because hNPs used for grafting here were cued to neuronal fates and any oligodendrocytes derived from such grafts might have been at a relatively immature state of differentiation. Our observation that, in contrast to the host, we rarely found human CNPase^+^ oligodendrocytes in transplants, is also supportive of the latter idea.

Neurons in ChR2-hNP transplants are predominantly GABAergic. The differentiation of NP grafts in important ways recapitulates neural development and GABA is the earliest neurotransmitter in CNS development, especially cortex, with widely ranging effects from progenitor proliferation to the migration and maturation of newborn neurons [55]. The fact that ChR2-hNPs were cued to forebrain/cortical fates may have contributed to this phenotypic outcome which is also consistent with previous work from our lab showing that human NSC-derived neurons may have a generic propensity to develop GABAergic phenotypes *in vivo* [7, 10]. The subtype of GABAergic neurons into which ChR2-hNPs differentiate is an important issue that is beyond the scope of this paper.

At least based on c-FOS induction in transplant as well as host cortical neurons, the effects of optical stimulation were inconclusive. The predominantly inhibitory differentiation of hChR2 may be partially to blame: turning on GABAergic neurons obviously inhibits downstream neuronal targets and the extensive connections between interneurons and projection neurons within the cortical circuity but also the extensive interconnectivity between differentiated neurons within the transplant itself may have caused complex transsynaptic effects leading to downstream inhibition as well as excitation. Other challenges include the degree to which the light penetrated enough into the transplant to stimulate an adequate number of hChR2^+^ neurons and the potential subthreshold input of transplant-derived neurons on individual cortical neurons of the host. On the other hand, we found that a consistent response to optogenetic stimulation was increase in respiratory rate (Ziogas NK and Sima R, personal observations). This anecdotal observation may be related to the dense terminal fields of differentiated grafts in cingulate cortex, a paralimbic cortical region who has well known associations with breathing and respiratory drive [56–59]. Based on the survey of our material, we hypothesize that there are at least three types of c-FOS immunoreactivity inside the transplants: first, the primarily stimulated hChR2^+^ neurons that do not happen to be inhibited by concomitantly stimulated, differentiated neighbors; second, hChR2^+^ or hChR2^-^ cells innervated by inhibitory neurons that are themselves inhibited within local GABAergic circuits that abound in densely differentiated portions of transplants; third, immature cells with persisting mitotic activity in which c-FOS is activated in a tonic fashion that has nothing to do with light stimulation. This level of complexity makes the use of optogenetically endowed grafts as physiological means to modulate CNS circuits extremely difficult at this stage. One solution might be to use smaller grafts, for example with 30,000 hNPs or less [5] and place them within clearly defined pathways with relatively simple neurotransmitter signatures, for example in the putamen or globus pallidus in order to modulate cortico-striato-thalamic activity in models of movement disorders.

Despite challenges in characterizing optical stimulation effects *in vivo*, these experiments show that we now possess the basic methods to test the functional integration of neural stem cells of various origins into host neural systems with optogenetics. Experiments *in vitro* and *ex vivo* have been more successful than *in vivo* work. Still, showing that we can drive neural systems and affect function or behavior *in vivo* would be critical prior to considering potential clinical applications. Such applications may come in various forms, including novel neuromodulatory treatments with optogenetically drivable, strategically placed transplants which could modulate host neural circuits at the millisecond scale. Our work is generally supportive of this potential and also points to specific challenges and problems that would need to be tackled in the immediate future.

## Supporting information

S1 Fig Genomic DNA PCR amplification of hChR2 and YFP segments of H9 pLenti-EYFP-hChR2 clones. Transduced cells were dissociated as single cells and plated on MEF plate at low density. After 1–2 weeks, colonies were manually picked and transferred to a new MEF-free plate and then amplified. Cell pellets were collected to purify genomic DNA for subsequent PCR analysis to check for integrated YFP and hChR2 sequences using DirectPCR Lysis reagent (301-C, Viagen Biotech, Inc., LA) according to the manufacturer’s instructions. One-two μL of cell lysate were used for PCR to amplify hChR2 and YFP fragments. Primers (S1 Table) were used with HotStar Taq DNA polymerase (203203, Qiagen) with a cycling profile of 95°C for 15 min, 35 cycles (94°C, 15 sec; 55°C, 30 sec; 68°C, 60 sec) and 7 min at 68°C. Thirty % (19 out of 60) and 65% (38 out of 59) clones of colonies from moi = 1 and moi = 5 group, respectively, were positive for YFP and hChR2.
(TIF)

S2 Fig Co-localization of mature neuronal markers with hChR2-YFP in hChR2-hNP-derived neurons co-cultured with astrocytes. On Day 37, NPs were plated on CD1 astrocytes and fed with NDM every 2 days for 60 days. After fixation, cells were stained with the neuronal markers SYN1, vGLUT1, vGAT and TBR1 to explore colocalization with endogenous hChR2-YFP signals.
(TIF)

S3 Fig ChR2 expression in hChR2-hNP-derived neurons does not change their biophysical properties. (A) Human ChR2-expressing neurons visualized with phase-contrast or epifluorescence microscopy at 485 nm. Neuron in lower right is approached by a patch clamp electrode. (B) hChR2-neurons were maintained at -67 mV and step depolarized with 300 ms long voltage steps from -107 mV to + 83 mV in 10 mV increments. Current-Voltage relations show the presence of a high voltage-activated outward potassium current activating above -32.6 ± 2.6 mV in 80% of cells (n = 46) and a fast-activating and inactivating inward sodium current (I_Na_; arrow head) with a maximum amplitude of -2060 ± 256 pA and an activation threshold at– 36.6 ± 2.0 mV in 76% of cells (n = 46). Inset (B´) shows the sodium current response at an extended time axis. (C) Example recordings of spontaneous firing of APs recorded in current clamp from an hChR2^-^ neurons (top panel) and an hChR2^+^ neurons (bottom panel). For both cell types an individual AP is shown at an extended time scale, indicating threshold and half-width. Resting membrane potentials of hChR2^+^ hNP-derived neurons were– 46.5 ± 24.0 mV (n = 36), similar to hChR2^-^ hNP-derived neurons (-48.13 ± 21.0 mV, n = 8; p = 0.86, Student’s t-test). (D) hChR2^-^ neurons and hChR2^+^ neurons display similar spontaneous AP firing rates, input resistances, AP thresholds and AP half widths (p > 0.05, Student t-test).
(TIF)

S4 Fig Further support for hChR2-hNP transplant differentiation in rat motor cortex. (A-B) These two high-power illustrations show example of the neuronal differentiation of hChR2-hNPs based on the expression of the cytoskeletal neuronal marker TUJ1 (A) and YFP, a marker for the optogene hChR2 (B). Scale bars: 10μm.
(TIF)

S5 Fig Detail of terminal field of hChR2-hNP-derived neurons (located in the transplant) in cingulate cortex. This preparation was dually stained for two transplant-selective markers (YFP for hChR2 and hSYP for human synaptophysin) and demonstrates both the dense terminal field and the extensive colocalization of the two markets within individual transplant-derived axons and their processes (double labeling is white here). Human synaptophysin immunoreactivity is present both in axons and what appear to be individual synaptic profiles. Panels at bottom are magnifications of numbered areas in main panel. Panel (1) is also used for the composition of **Fig 10C**. Top of cortex is on the left. Scale bars: 50μm.
(TIF)

S6 Fig Phenotypic disposition of hChR2-hNP transplant with respect to inhibitory (GABA) or excitatory (glutamate) neurotransmission. **Illustrations are larger magnifications of panels A-B of Fig 11 and provide much greater detail.** These are representative illustrations from dually immunostained preparations for YFP (a hChR2 marker specific for the transplant and transplant-derived structures) and either vGAT (A), a presynaptic marker of GABAergic neurotransmission or vGLUT1 (B), a presynaptic marker of glutamatergic neurotransmission. Further explanation is given in the legend of **Fig 11**. Scale bars: 100μm.
(TIF)

S7 Fig Colocalization of a marker of GABAergic terminals, vGAT, in transplant-derived hSYP^+^ terminals in rat motor cortex. **This is the source confocal image that was magnified further to generate Fig 11C and 11C´.** There are many double labeled (yellow) profiles, with concentrations in regions indicated with numbers. Images in **Fig 11C and 11C´** are magnifications of regions 1 and 2. Scale bar: 20μm.
(TIF)

S8 Fig **A dually stained preparation with antibodies for YFP (for hChR2**^**+**^ **axons) and MBP (for myelin) through the border of a transplant as in Fig 12 with transversely (A, C) obliquely (B) and longitudinally (C) cut myelinated axons.** Most myelinated profiles are large (arrows in A-B, large arrow in C), but there are exceptional smaller axons (small arrows in C). Images are taken with a confocal microscope (single optical sections). Scale bars: 5μm.
(TIF)

S9 Fig Triply immunostained preparations with various combinations of antibodies against YFP (for hChR2^+^ axons), MBP (for myelin), Olig2 (for oligodendrocytes) and SC121 (for human graft identity) through the border of a transplant or through the adjacent motor cortex. Images were taken with a confocal microscope (single optical sections) and were chosen to illustrate the anatomical disposition of transplant-derived (human) oligodendrocytes in reference to transplant location. Oligodendrocytes are in red, human identities are labeled white and transplant-derived axons are in green. Section is from a case with a relatively greater number of graft-derived oligodendrocytes (note that differentiation was predominantly neuronal in all cases). A is taken just at the border of the graft, B corresponds to host (rat) cortex just outside the caudal end the graft and C is taken from a field deeply into host (rat) cortex. Observe the absence of transplant-derived (white) oligodendrocytes in B and C. Nuclei are stained with DAPI (blue). Scale bars: A-C, 20μm.
(TIF)

S10 Fig A triply immunostained preparation with antibodies against YFP (for hChR2^+^ axons), MBP (for myelin) and SC121 (for human graft identity) through the border of a transplant. Images were captured with a confocal microscope (single optical sections) and illustrate contacting activity occasionally displayed by some human oligodendrocytes at the border of the graft. Myelin is in red, hChR2^+^ axons in green, and human cell identities are labeled white. B-E are magnifications of numbered loci in panel A, added here for greater cellular detail. Main panel features a classically appearing oligodendrocyte profile of human origin (arrow, white) sending horizontal and vertical processes to contact human (green) and perhaps non-human axons at the border of the graft. Note the presence of SC121 immunoreactivity in some myelin sheaths or in unmyelinated YFP^+^ axons. In E, observe the characteristic stepwise contact when the oligodendrocytic process encounters axons. Nuclei are stained with DAPI (blue). Scale bars: A, 10μm; B-E, 5μm.
(TIF)

S11 Fig An example of c-FOS activity in a stimulated hChR2-hNP transplant (Case R3): This is a c-FOS active graft, with uneven differentiation, one hour after stimulation. Together with other examples illustrated in **S12** and **S13 Figs**, figure showcases the difficulty in drawing conclusions on the functionality of transplant-derived synaptic inputs on host neurons (as established on the basis of changes in c-FOS immunoreactivity in host neurons after optic stimulation). Images here and in **S12** and **S13 Figs** were captured with epifluorescence microscopy. A and B are from different sites within the graft. From top to bottom, the same field is visualized with blue filter for graft-derived terminals (hSYP, green), green filter for c-FOS^+^ nuclei (red), and UV filter for cellularity and gross nuclear maturity with DAPI (blue) or images were combined to showcase co-labeling of hSYP with c-FOS or c-FOS with DAPI. Islands of c-FOS immunoreactivity avoid synaptically dense areas as well as small areas of immature cells which are indicated with asterisks in A and B, although there is rare confluent c-FOS immunoreactivity in some neuroblasts (not shown). Scale bars: 50μm.
(TIF)

S12 Fig Another examples of c-FOS activity in a stimulated hChR2-hNP transplant (Case R4): This is a c-FOS inactive graft, with very good homogeneous differentiation, one hour after stimulation. From top to bottom, the same field through the graft is visualized with blue filter for graft-derived terminals (hSYP, green), green filter for c-FOS+ nuclei (red), and UV filter for cellularity and gross nuclear maturity with DAPI (blue) or images were combined as in S11 Fig. Transplant is c-FOS negative throughout, despite optic stimulation. Scale bars: 100μm.
(TIF)

S13 Fig An example of c-FOS activity in an unstimulated hChR2-hNP transplants (Case R5): This is a c-FOS active graft, with uneven differentiation. From top to bottom, the same field through the graft is visualized essentially as **S11** and **S12 Figs** minus DAPI counterstain. There is extensive c-FOS immunoreactivity in synaptically dense areas of the graft and also in the neighboring host cortex. Scale bars: 50μm.
(TIF)

S14 Fig **A confocal image (single optical section) taken from the section illustrated in S7 and S11 Figs (Case R3) to showcase the higher density of c-FOS**^**+**^ **nuclei (red) in regions of low human synaptic density (center) in contrast to high synaptic density (hSYP; green, top left and lower right).** Scale bar: 20μm.
(TIF)

S1 Table Primers used for genomic DNA PCR to detect integrated channelrhodopsin (ChR2) and YFP genes. (PDF)

S2 Table Antibodies used in research related to this paper. (DOCX)
