# Supplementary figures and images for: Optogenetically transduced human ES cell-derived neural progenitors and their neuronal progenies: Phenotypic characterization and responses to optical stimulation
Source: PLoS One. 2019 Nov 11;14(11):e0224846. doi: 10.1371/journal.pone.0224846 (PMC6844486; doi:10.1371/journal.pone.0224846)

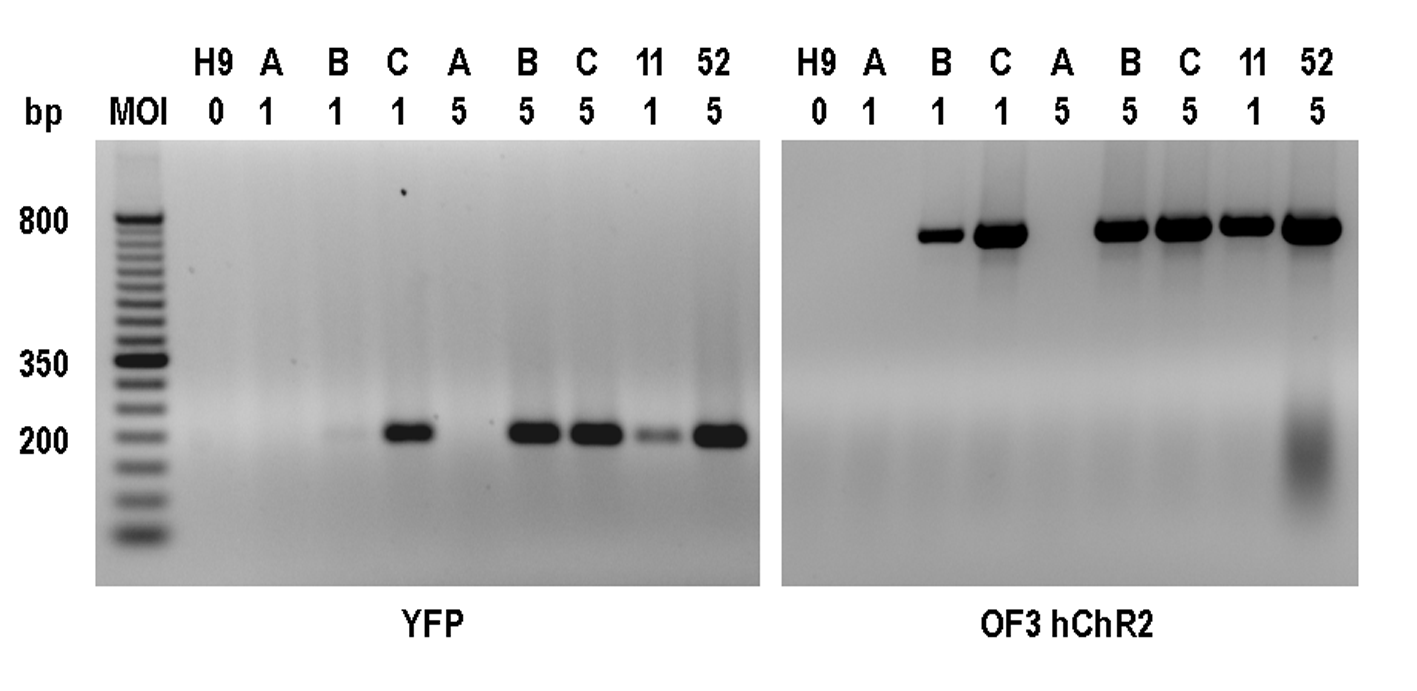

Supplement: S1 Fig — Transduced cells were dissociated as single cells and plated on MEF plate at low density. After 1–2 weeks, colonies were manually picked and transferred to a new MEF-free plate and then amplified. Cell pellets were collected to purify genomic DNA for subsequent PCR analysis to check for integrated YFP and hChR2 sequences using DirectPCR Lysis reagent (301-C, Viagen Biotech, Inc., LA) according to the manufacturer’s instructions. One-two μL of cell lysate were used for PCR to amplify hChR2 and YFP fragments. Primers (S1 Table) were used with HotStar Taq DNA polymerase (203203, Qiagen) with a cycling profile of 95°C for 15 min, 35 cycles (94°C, 15 sec; 55°C, 30 sec; 68°C, 60 sec) and 7 min at 68°C. Thirty % (19 out of 60) and 65% (38 out of 59) clones of colonies from moi = 1 and moi = 5 group, respectively, were positive for YFP and hChR2. (TIF) [file pone.0224846.s001.tif]

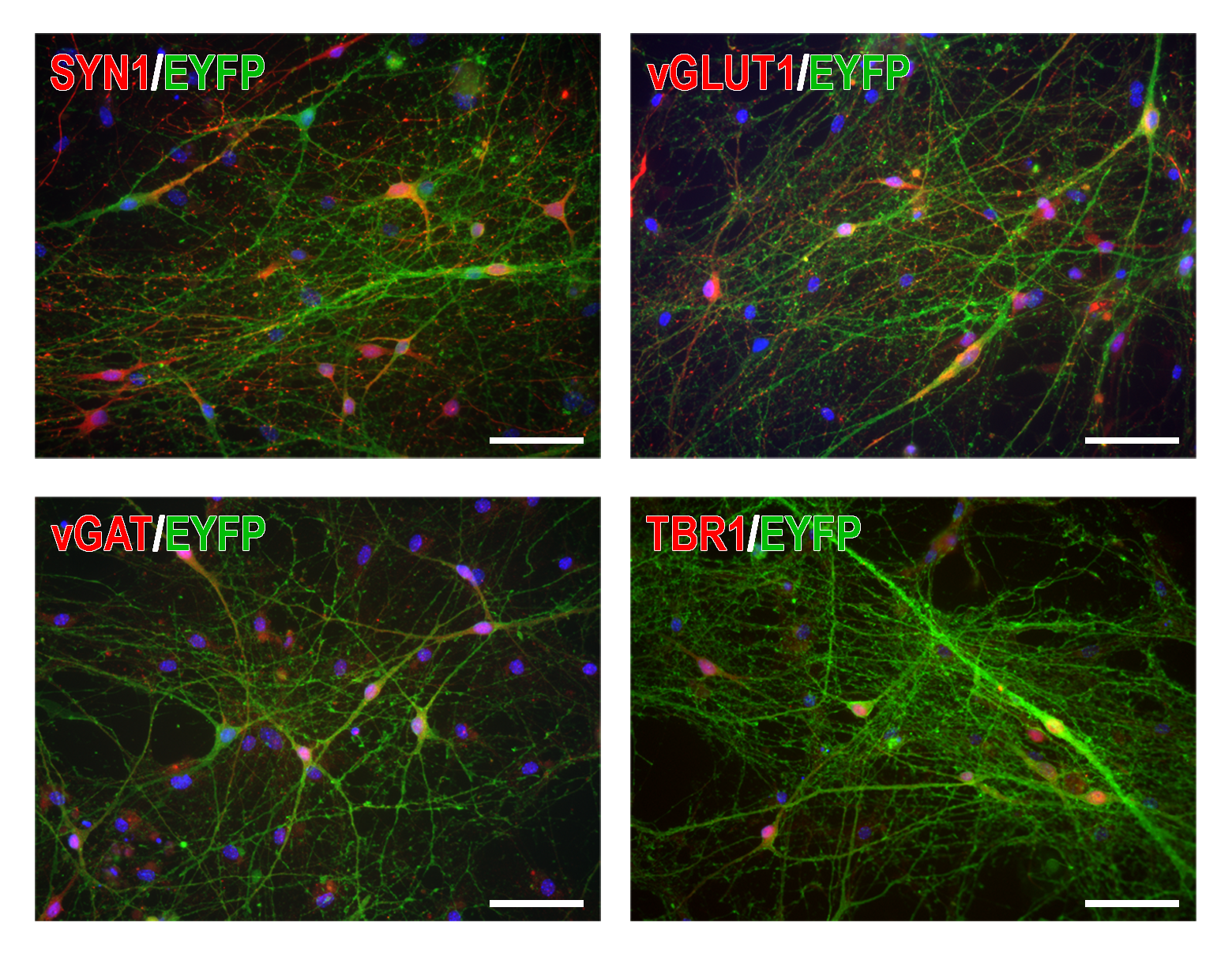

Supplement: S2 Fig — On Day 37, NPs were plated on CD1 astrocytes and fed with NDM every 2 days for 60 days. After fixation, cells were stained with the neuronal markers SYN1, vGLUT1, vGAT and TBR1 to explore colocalization with endogenous hChR2-YFP signals. (TIF) [file pone.0224846.s002.tif]

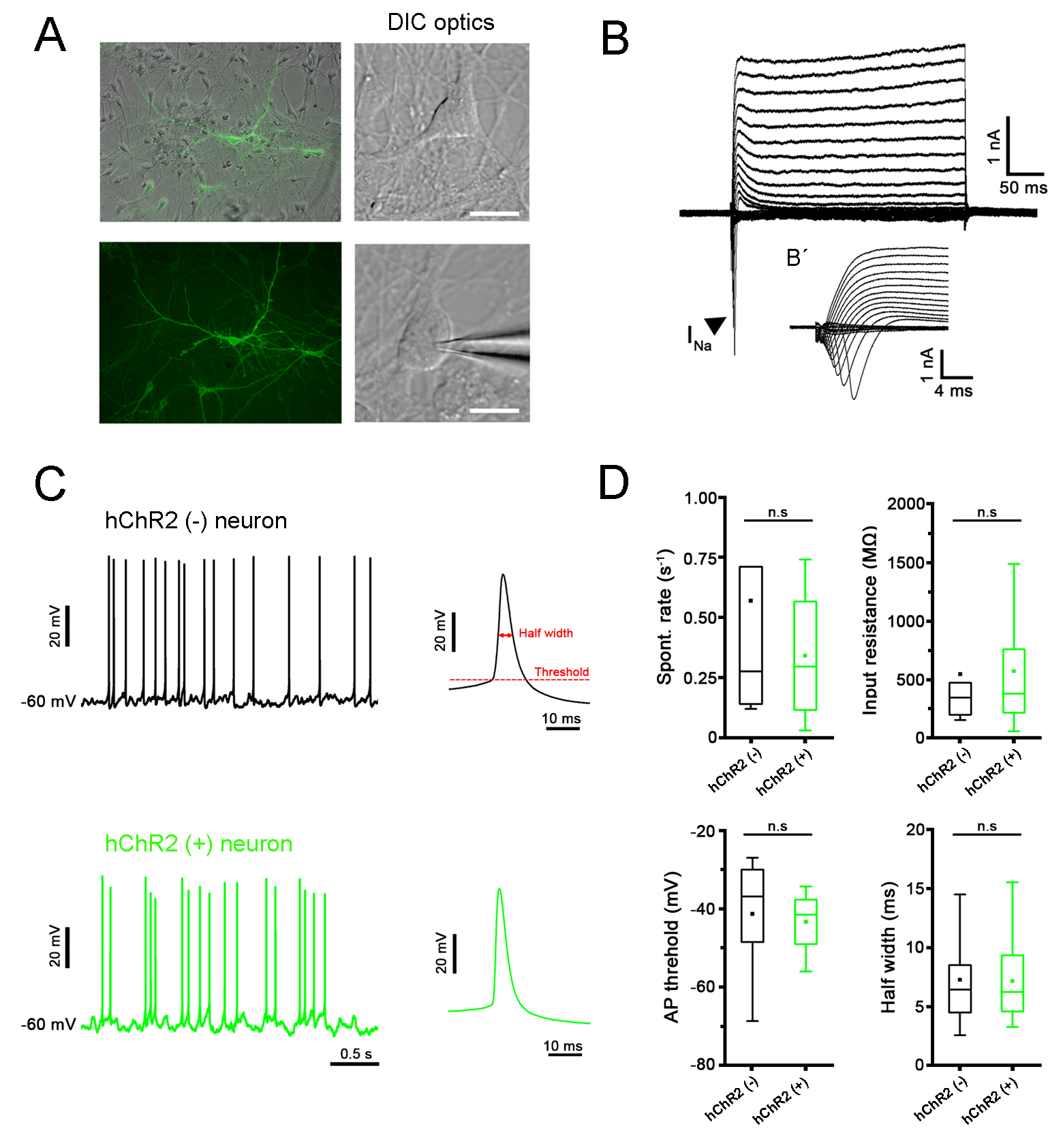

Supplement: S3 Fig — (A) Human ChR2-expressing neurons visualized with phase-contrast or epifluorescence microscopy at 485 nm. Neuron in lower right is approached by a patch clamp electrode. (B) hChR2-neurons were maintained at -67 mV and step depolarized with 300 ms long voltage steps from -107 mV to + 83 mV in 10 mV increments. Current-Voltage relations show the presence of a high voltage-activated outward potassium current activating above -32.6 ± 2.6 mV in 80% of cells (n = 46) and a fast-activating and inactivating inward sodium current (INa; arrow head) with a maximum amplitude of -2060 ± 256 pA and an activation threshold at– 36.6 ± 2.0 mV in 76% of cells (n = 46). Inset (B´) shows the sodium current response at an extended time axis. (C) Example recordings of spontaneous firing of APs recorded in current clamp from an hChR2- neurons (top panel) and an hChR2+ neurons (bottom panel). For both cell types an individual AP is shown at an extended time scale, indicating threshold and half-width. Resting membrane potentials of hChR2+ hNP-derived neurons were– 46.5 ± 24.0 mV (n = 36), similar to hChR2- hNP-derived neurons (-48.13 ± 21.0 mV, n = 8; p = 0.86, Student’s t-test). (D) hChR2- neurons and hChR2+ neurons display similar spontaneous AP firing rates, input resistances, AP thresholds and AP half widths (p > 0.05, Student t-test). (TIF) [file pone.0224846.s003.tif]

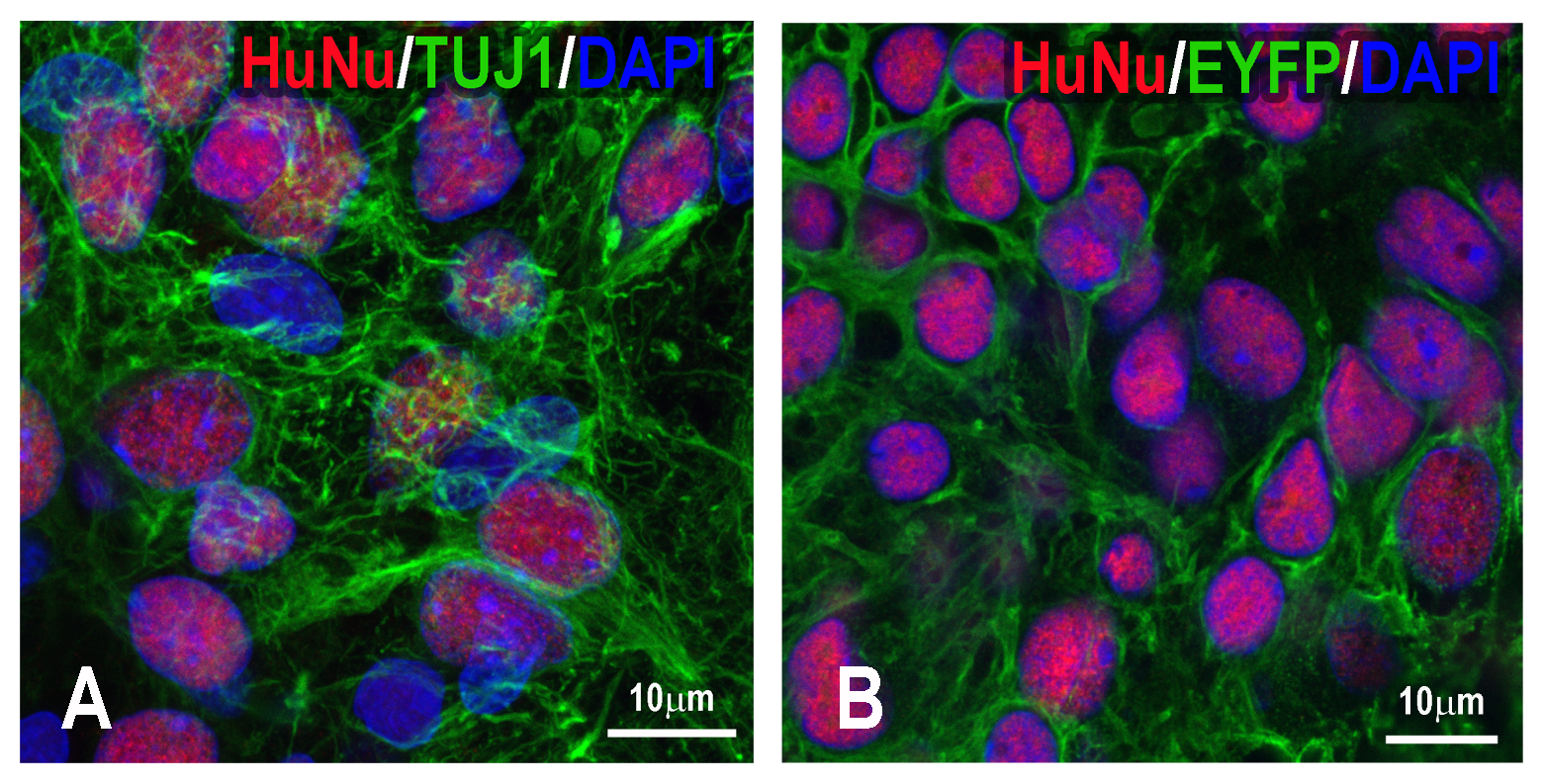

Supplement: S4 Fig — (A-B) These two high-power illustrations show example of the neuronal differentiation of hChR2-hNPs based on the expression of the cytoskeletal neuronal marker TUJ1 (A) and YFP, a marker for the optogene hChR2 (B). Scale bars: 10μm. (TIF) [file pone.0224846.s004.tif]

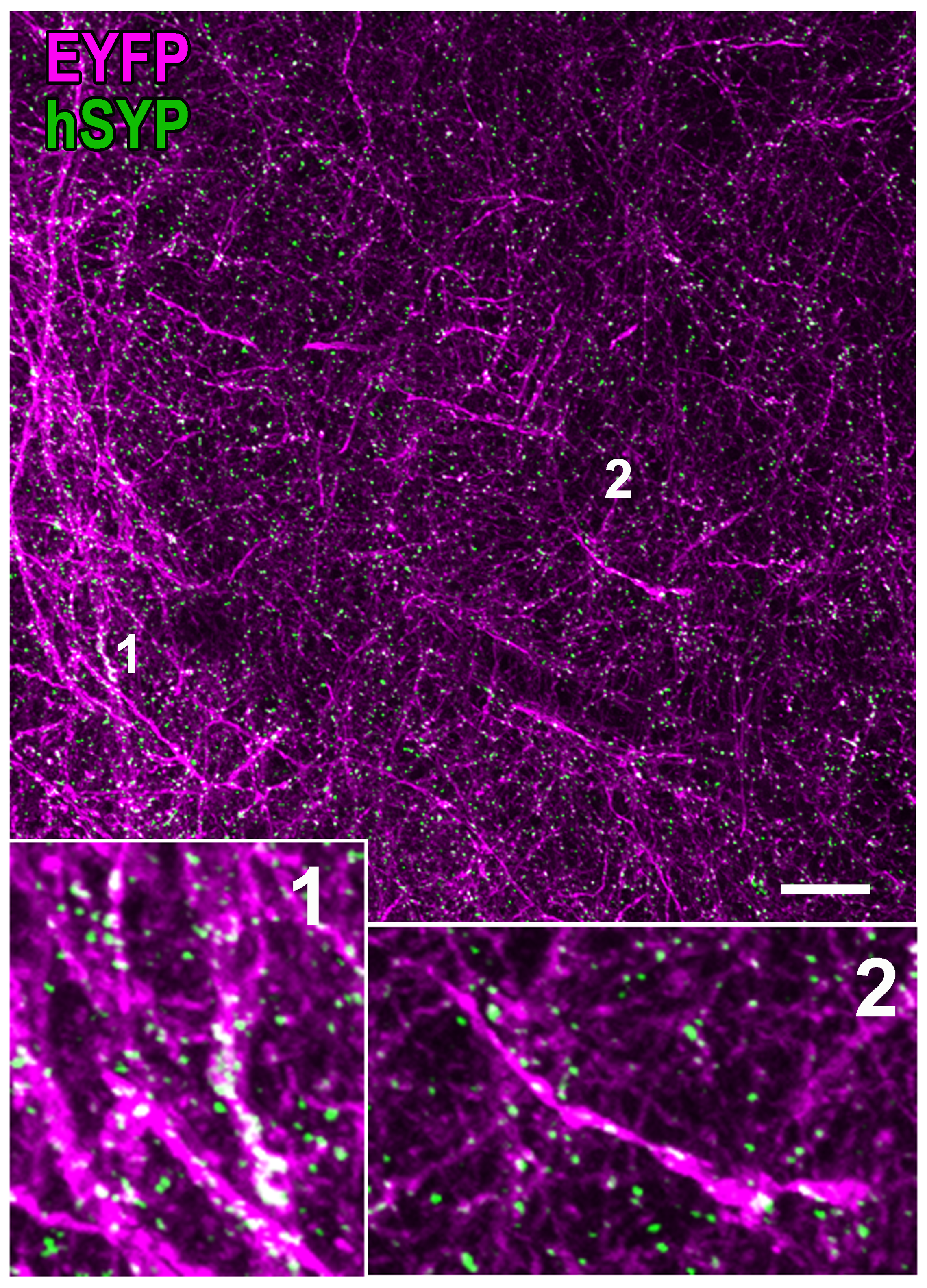

Supplement: S5 Fig — This preparation was dually stained for two transplant-selective markers (YFP for hChR2 and hSYP for human synaptophysin) and demonstrates both the dense terminal field and the extensive colocalization of the two markets within individual transplant-derived axons and their processes (double labeling is white here). Human synaptophysin immunoreactivity is present both in axons and what appear to be individual synaptic profiles. Panels at bottom are magnifications of numbered areas in main panel. Panel (1) is also used for the composition of Fig 10C. Top of cortex is on the left. Scale bars: 50μm. (TIF) [file pone.0224846.s005.tif]

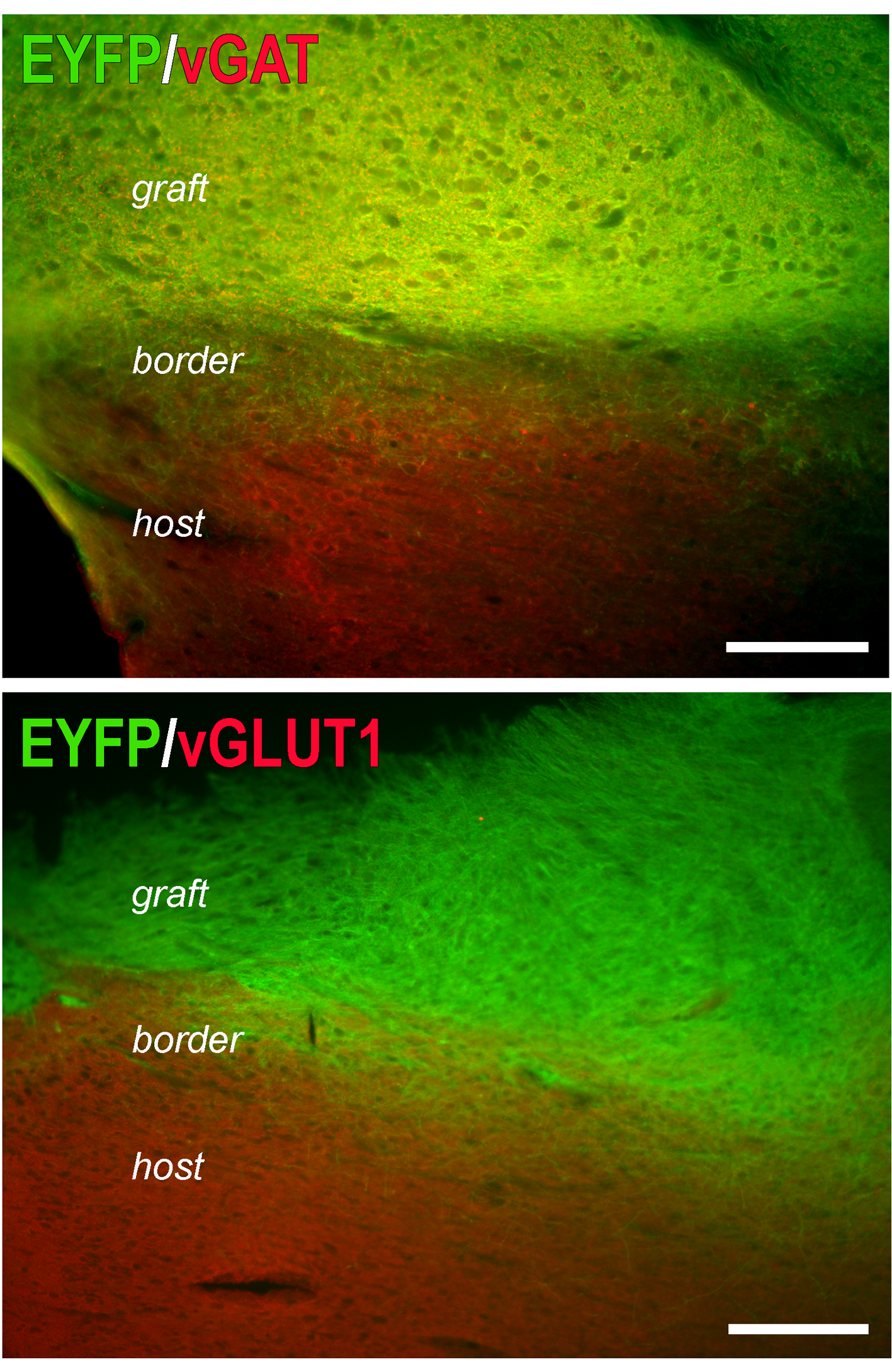

Supplement: S6 Fig — Illustrations are larger magnifications of panels A-B of Fig 11 and provide much greater detail. These are representative illustrations from dually immunostained preparations for YFP (a hChR2 marker specific for the transplant and transplant-derived structures) and either vGAT (A), a presynaptic marker of GABAergic neurotransmission or vGLUT1 (B), a presynaptic marker of glutamatergic neurotransmission. Further explanation is given in the legend of Fig 11. Scale bars: 100μm. (TIF) [file pone.0224846.s006.tif]

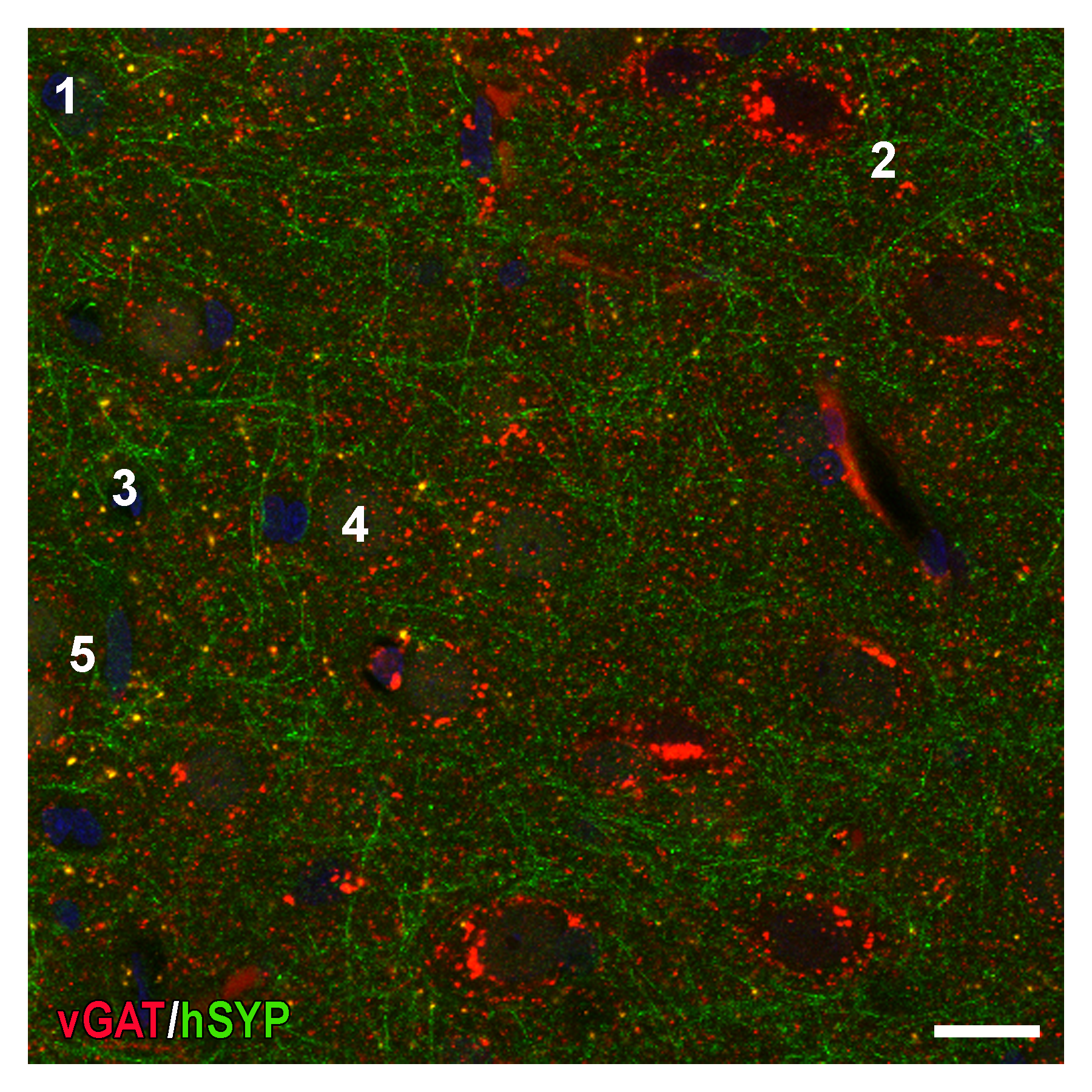

Supplement: S7 Fig — This is the source confocal image that was magnified further to generate Fig 11C and 11C´. There are many double labeled (yellow) profiles, with concentrations in regions indicated with numbers. Images in Fig 11C and 11C´ are magnifications of regions 1 and 2. Scale bar: 20μm. (TIF) [file pone.0224846.s007.tif]

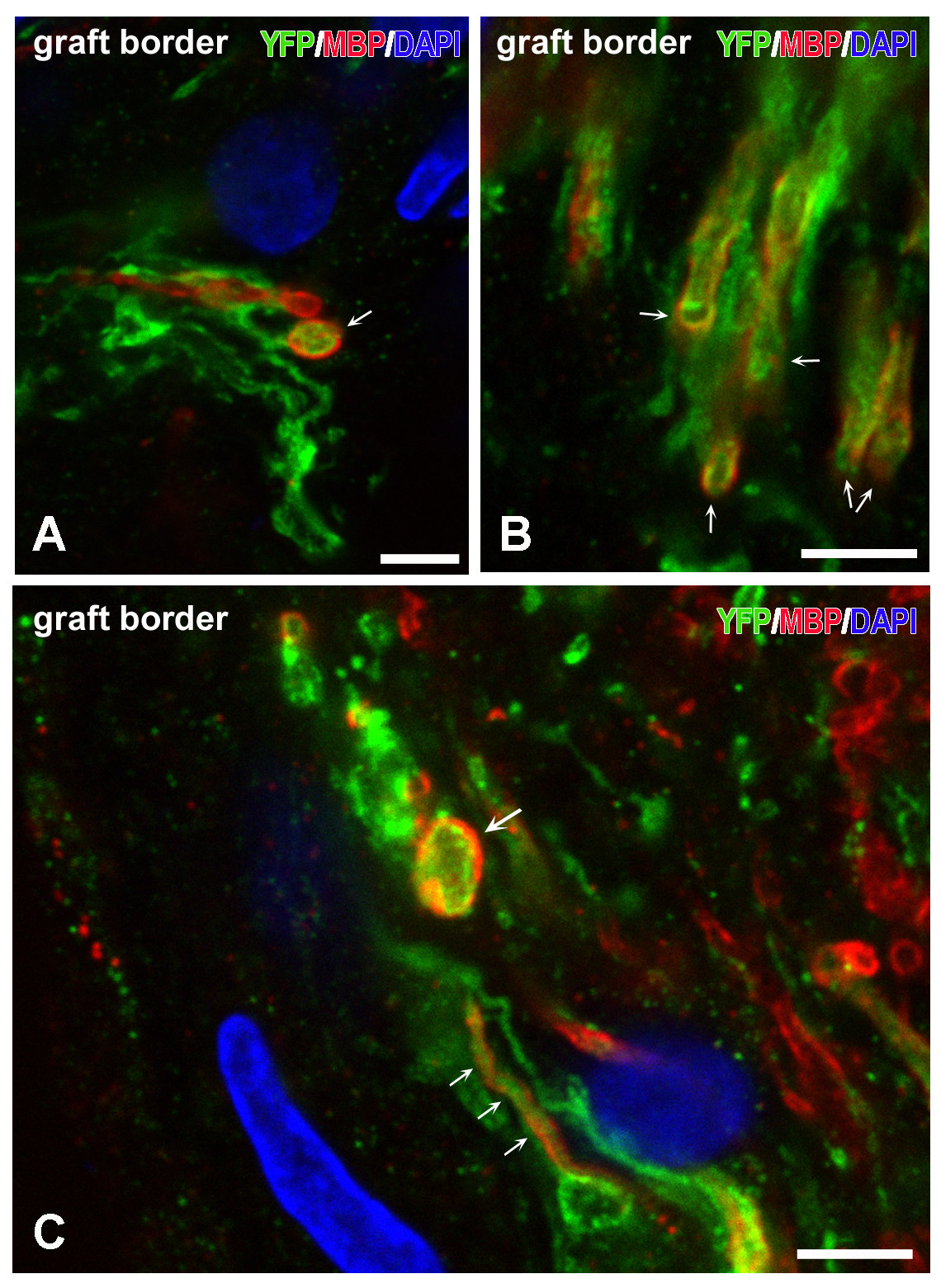

Supplement: S8 Fig — A dually stained preparation with antibodies for YFP (for hChR2+ axons) and MBP (for myelin) through the border of a transplant as in Fig 12 with transversely (A, C) obliquely (B) and longitudinally (C) cut myelinated axons. Most myelinated profiles are large (arrows in A-B, large arrow in C), but there are exceptional smaller axons (small arrows in C). Images are taken with a confocal microscope (single optical sections). Scale bars: 5μm. (TIF) [file pone.0224846.s008.tif]

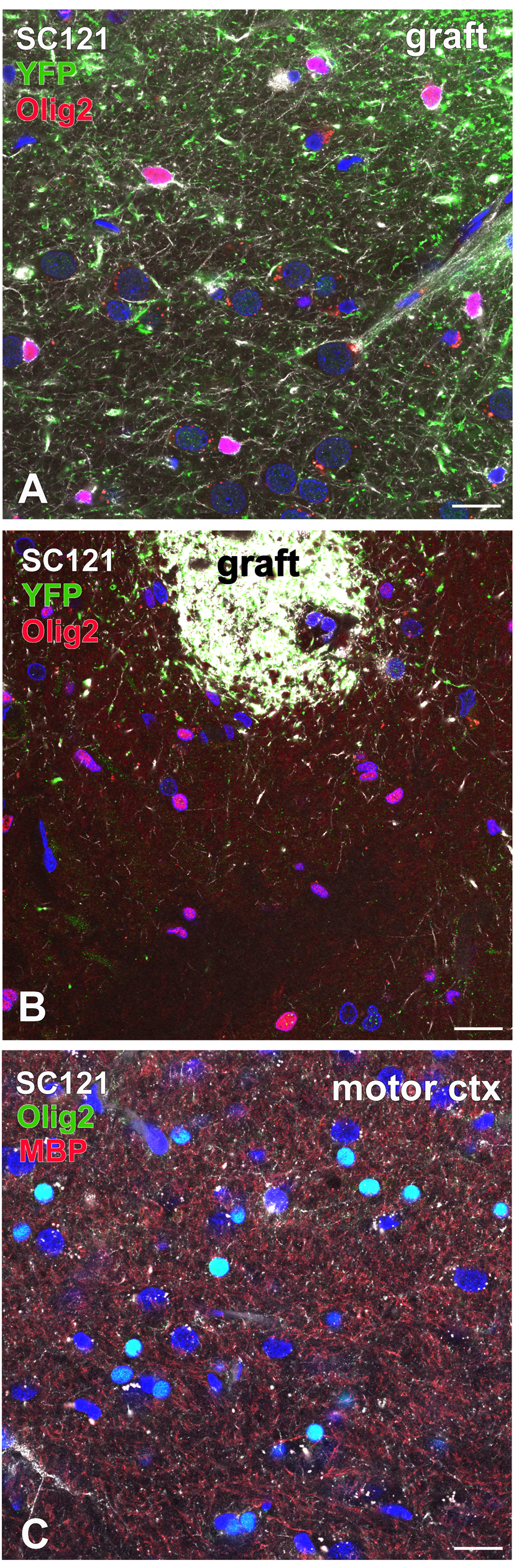

Supplement: S9 Fig — Images were taken with a confocal microscope (single optical sections) and were chosen to illustrate the anatomical disposition of transplant-derived (human) oligodendrocytes in reference to transplant location. Oligodendrocytes are in red, human identities are labeled white and transplant-derived axons are in green. Section is from a case with a relatively greater number of graft-derived oligodendrocytes (note that differentiation was predominantly neuronal in all cases). A is taken just at the border of the graft, B corresponds to host (rat) cortex just outside the caudal end the graft and C is taken from a field deeply into host (rat) cortex. Observe the absence of transplant-derived (white) oligodendrocytes in B and C. Nuclei are stained with DAPI (blue). Scale bars: A-C, 20μm. (TIF) [file pone.0224846.s009.tif]

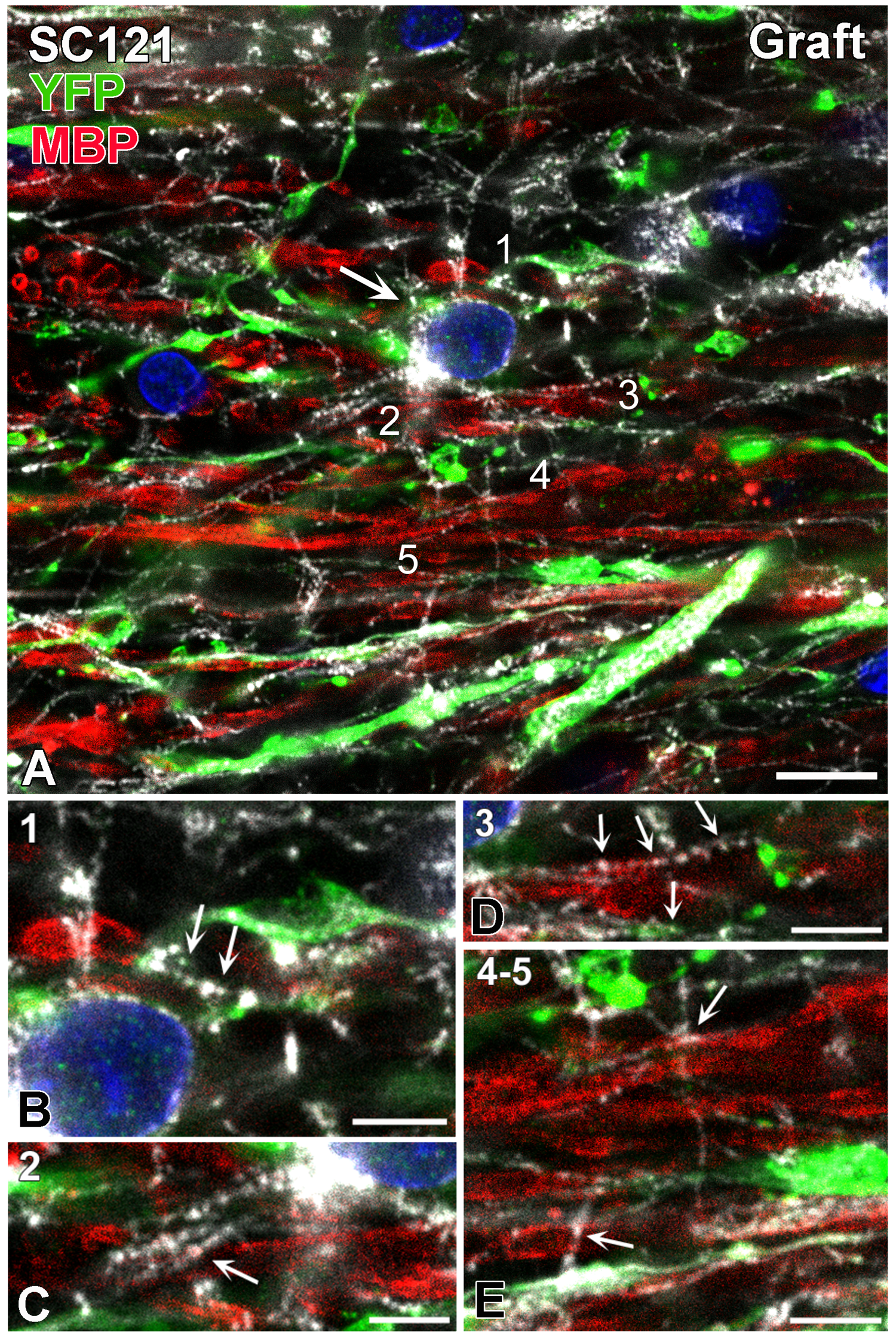

Supplement: S10 Fig — Images were captured with a confocal microscope (single optical sections) and illustrate contacting activity occasionally displayed by some human oligodendrocytes at the border of the graft. Myelin is in red, hChR2+ axons in green, and human cell identities are labeled white. B-E are magnifications of numbered loci in panel A, added here for greater cellular detail. Main panel features a classically appearing oligodendrocyte profile of human origin (arrow, white) sending horizontal and vertical processes to contact human (green) and perhaps non-human axons at the border of the graft. Note the presence of SC121 immunoreactivity in some myelin sheaths or in unmyelinated YFP+ axons. In E, observe the characteristic stepwise contact when the oligodendrocytic process encounters axons. Nuclei are stained with DAPI (blue). Scale bars: A, 10μm; B-E, 5μm. (TIF) [file pone.0224846.s010.tif]

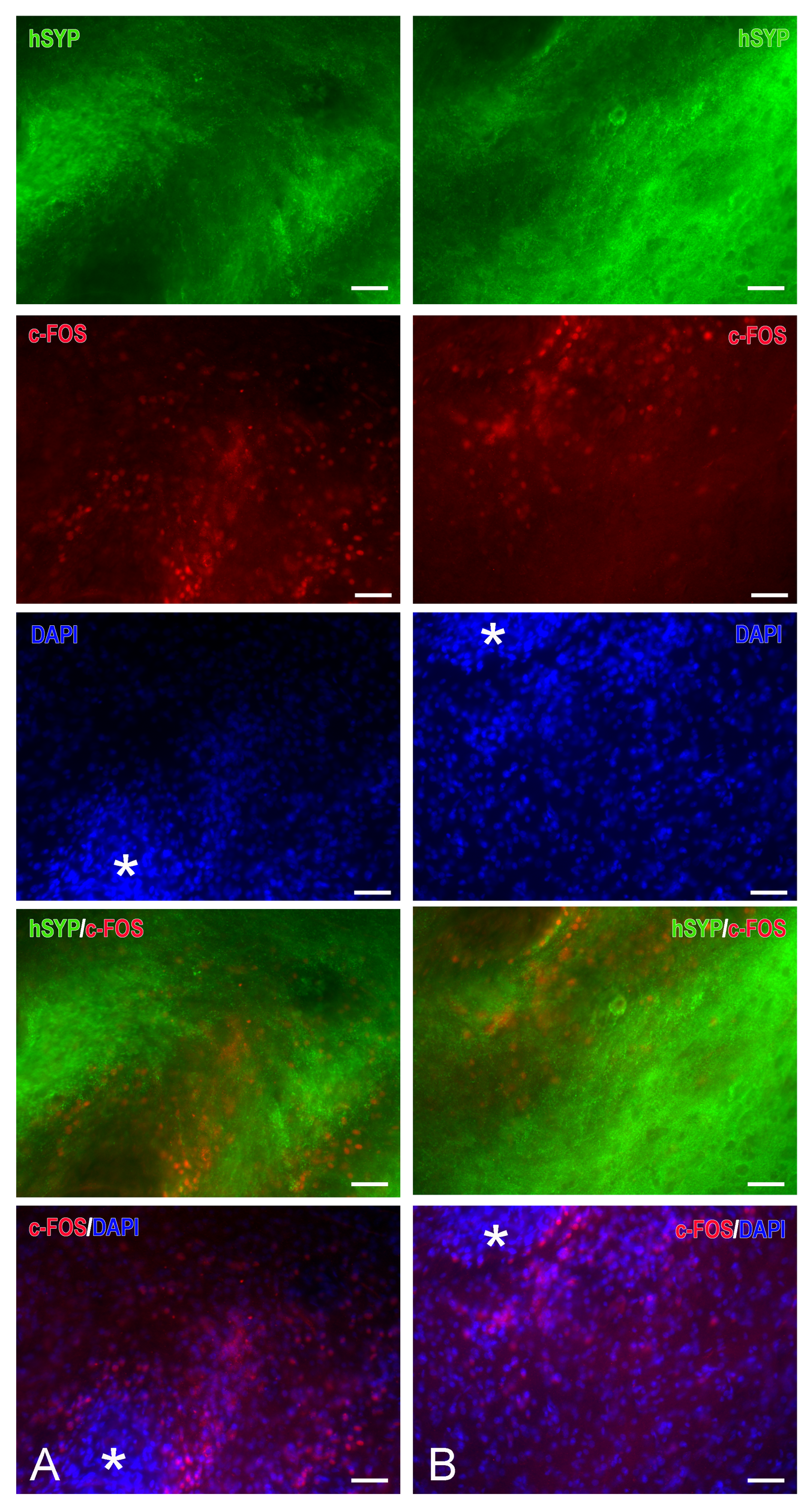

Supplement: S11 Fig — Together with other examples illustrated in S12 and S13 Figs, figure showcases the difficulty in drawing conclusions on the functionality of transplant-derived synaptic inputs on host neurons (as established on the basis of changes in c-FOS immunoreactivity in host neurons after optic stimulation). Images here and in S12 and S13 Figs were captured with epifluorescence microscopy. A and B are from different sites within the graft. From top to bottom, the same field is visualized with blue filter for graft-derived terminals (hSYP, green), green filter for c-FOS+ nuclei (red), and UV filter for cellularity and gross nuclear maturity with DAPI (blue) or images were combined to showcase co-labeling of hSYP with c-FOS or c-FOS with DAPI. Islands of c-FOS immunoreactivity avoid synaptically dense areas as well as small areas of immature cells which are indicated with asterisks in A and B, although there is rare confluent c-FOS immunoreactivity in some neuroblasts (not shown). Scale bars: 50μm. (TIF) [file pone.0224846.s011.tif]

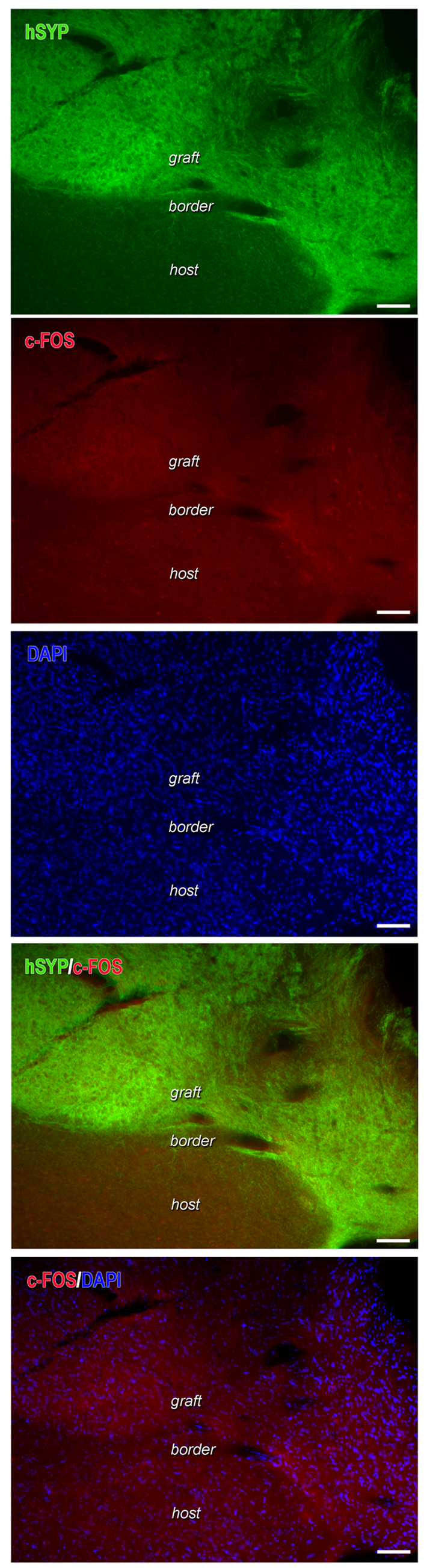

Supplement: S12 Fig — From top to bottom, the same field through the graft is visualized with blue filter for graft-derived terminals (hSYP, green), green filter for c-FOS+ nuclei (red), and UV filter for cellularity and gross nuclear maturity with DAPI (blue) or images were combined as in S11 Fig. Transplant is c-FOS negative throughout, despite optic stimulation. Scale bars: 100μm. (TIF) [file pone.0224846.s012.tif]

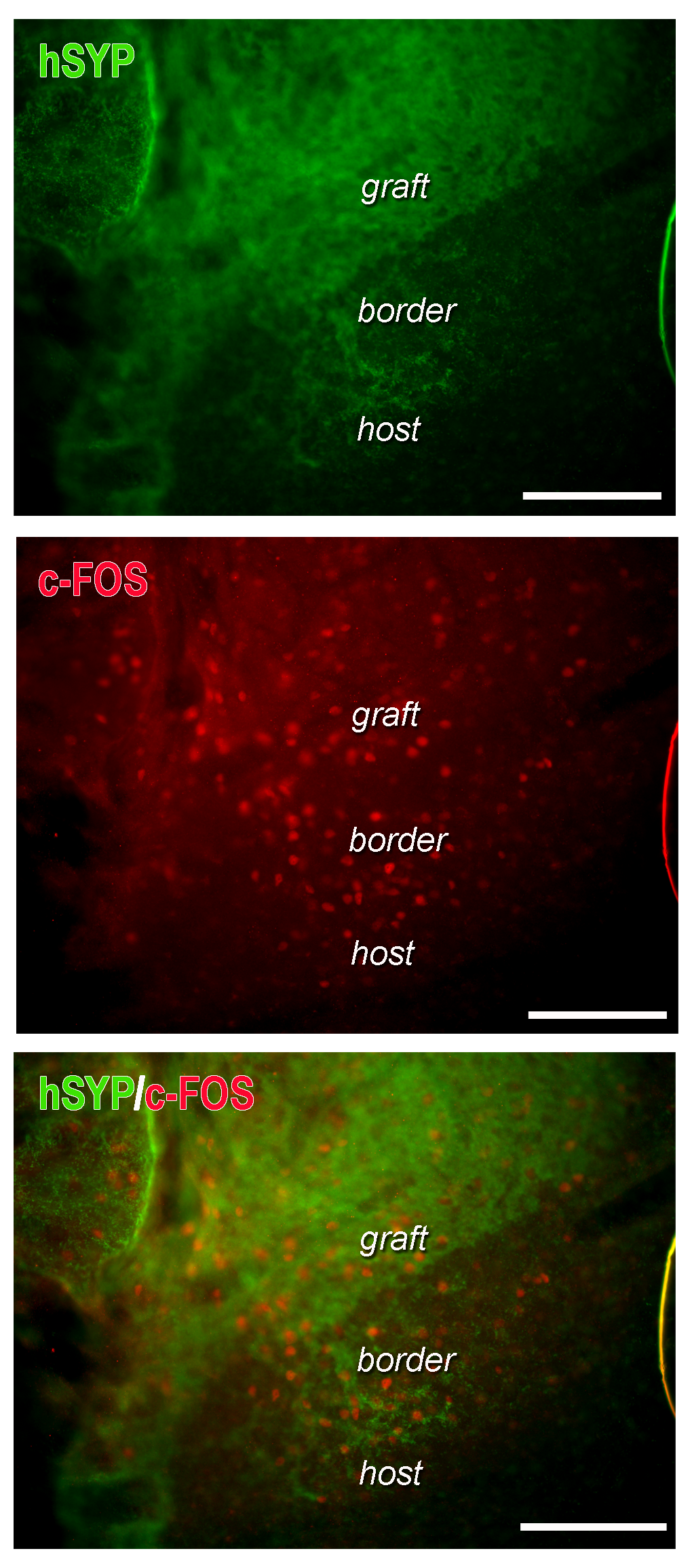

Supplement: S13 Fig — From top to bottom, the same field through the graft is visualized essentially as S11 and S12 Figs minus DAPI counterstain. There is extensive c-FOS immunoreactivity in synaptically dense areas of the graft and also in the neighboring host cortex. Scale bars: 50μm. (TIF) [file pone.0224846.s013.tif]

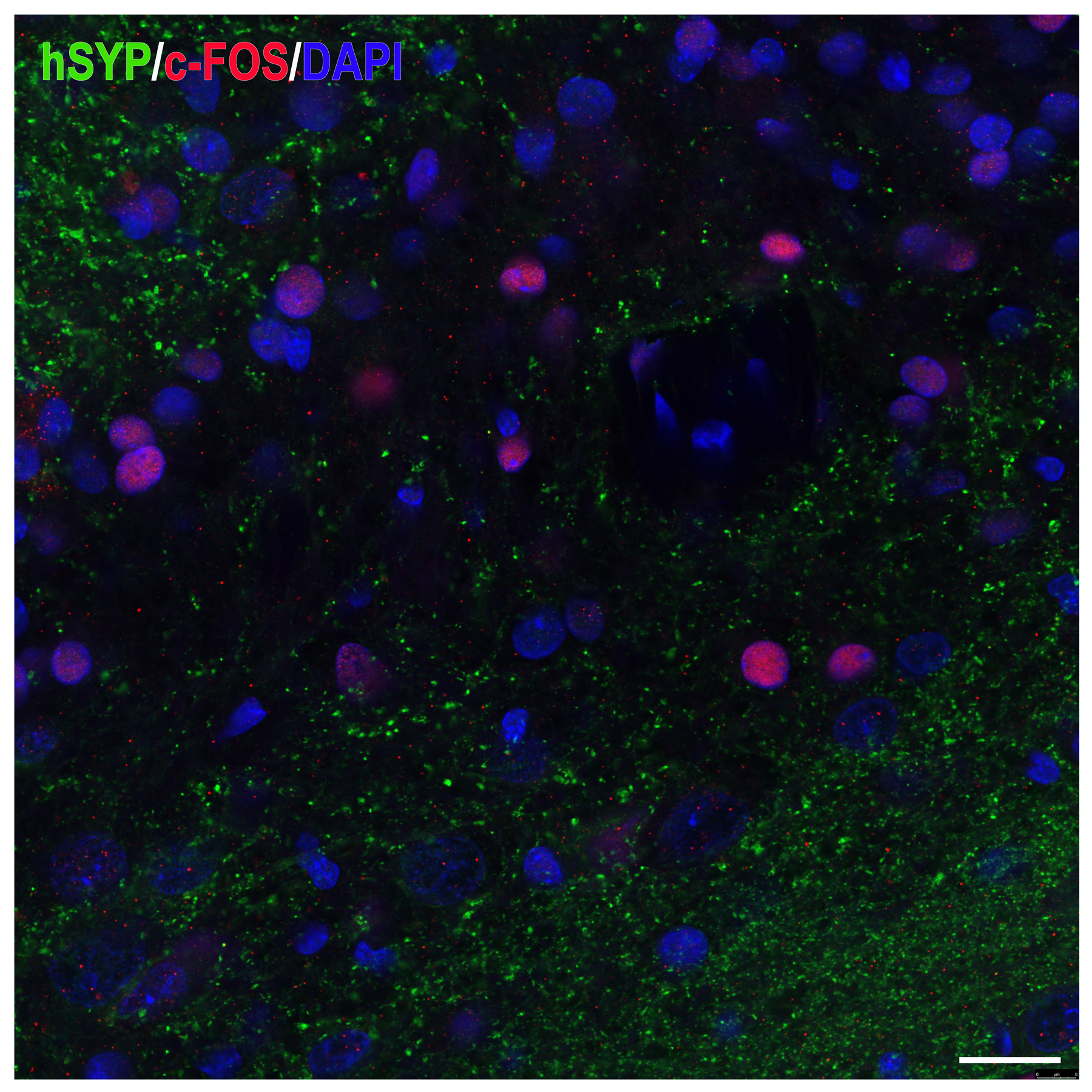

Supplement: S14 Fig — A confocal image (single optical section) taken from the section illustrated in S7 and S11 Figs (Case R3) to showcase the higher density of c-FOS+ nuclei (red) in regions of low human synaptic density (center) in contrast to high synaptic density (hSYP; green, top left and lower right). Scale bar: 20μm. (TIF) [file pone.0224846.s014.tif]
