# Supplementary material for: Optogenetically transduced human ES cell-derived neural progenitors and their neuronal progenies: Phenotypic characterization and responses to optical stimulation
Source: PLoS One. 2019 Nov 11;14(11):e0224846. doi: 10.1371/journal.pone.0224846 (PMC6844486; doi:10.1371/journal.pone.0224846)
Supplement: S1 Table — (PDF) [file pone.0224846.s015.pdf]

Table S1. Primers used for genomic DNA PCR to detect integrated channelrhodopsin (ChR2) and eYFP genes.

Primers	Sequences (5’ to 3’)
EYFP-FW	ACGTAAACGGCCACAAGTTC
EYFP-RV	AAGTCGTGCTGCTTCATGTG
SP PLENTI-SYN (ChR2)	GGTCCCTGAGGATCAATG
AS PLENTI-SYN (ChR2)	TACGGTGGAGCCATAGACG
