# Supplementary material for: Optogenetically transduced human ES cell-derived neural progenitors and their neuronal progenies: Phenotypic characterization and responses to optical stimulation
Source: PLoS One. 2019 Nov 11;14(11):e0224846. doi: 10.1371/journal.pone.0224846 (PMC6844486; doi:10.1371/journal.pone.0224846)
Supplement: S2 Table — (DOCX) [file pone.0224846.s016.docx]

Table S2. Antibodies used in research related to this paper.

| Antigen | Full Name | Marker for | Host | Dilution | Catalogue # | Vendor |
| --- | --- | --- | --- | --- | --- | --- |
| BMP4 | Bone morphogenetic protein 4 | Mesodermal lineage | Rabbit | 1:200 | ab39973 | Abcam |
| BRN2 | POU Class 3 Homeobox 2 | Progenitor cells and Upper Layer II, III and V cortical neurons | Rabbit | 1:500 | PA5-30124 | Thermo Scientific |
| CNPase | 2',3'-cyclic-nucleotide 3'-phosphodiesterase | Myelinating Oligodendrocytes | Rabbit | 1:500 | 355-003 | Synaptic System |
| CTIP2 | COUP-TF-Interacting Protein 2 | Layer V and VI cortical neurons | Rabbit | 1:200 | ab28448 | Abcam |
| cFOS | Proto-oncogene vFos homolog | Immediate early gene (IEG) marker | Rabbit | 1:500 | 226-003 | Synaptic Systems |
| DCX | Doublecortin | Migrating early neuronal cells | Rabbit | 1:400 | 4604 | Cell signaling technology |
| EYFP | Enhanced yellow fluorescent protein | Optogenetic reporter | Rabbit | 1:200 | MBS8500431 | eBiosciences |
| FOXG1 | Forkhead Box G1 | Primary progenitor, Telencephalon | Rabbit | 1:200 | ab18259 | Abcam |
| GABA | Gamma aminobutyric acid | Inhibitory neurotransmitter | Guinea pig | 1:500 | ab17413 | Abcam |
| GAD1/GAD67 | Glutamate decarboxylase 1 | Mature inhibitory GABAergic neurons | Rabbit | 1:500 | 198-013 | Synaptic Systems |
| GFAP | Glial fibrillary acidic protein | Astrocytes | Rabbit | 1:500 | 04-1031 | Millipore |
| GFP | Green fluorescent protein | Optogenetic reporter | Chicken | 1:500 | ab13970 | Abcam |
| Ki67 | Antigen Ki-67 | Mitotic proliferating cells | Rabbit | 1:400 | ab15580 | Abcam |
| MAP2 | Microtubule associated protein 2 | Mature neurons | Mouse | 1:500 | MAB3418 | Millipore |
| MBP | Myelin basic protein | Oligodendrocyte myelin basic protein | Rabbit | 1:500 | Ab40390 | Abcam |
| Nestin | Nestin, Intermediate filament protein (class type VI) | Primary progenitor | Rabbit | 1:500 | ABD69 | Millipore |
| OCT4 | Octamer-binding transcription factor 4 (POU5F1) | Stem cells (undifferentiated) | Mouse | 1:200 | 611202 | BD Bioscience |
| Olig2 | Oligodendrocyte transcription factor 2 | Oligodendrocyte nuclear marker | Goat | 1:200 | AF2418 | R&D Systems |
| Olig2 | Oligodendrocyte transcription factor 2 | Oligodendrocyte nuclear marker | Rabbit | 1:200 | Ab109186 | Abcam |
| PAX6 | Paired box protein Pax-6 | Neural progenitor | Rabbit | 1:200 | 09-0075 | Stemgent |
| PDGFRα | Platelet-derived growth factor receptor alpha | Oligodendrocytes | Rabbit | 1:200 | sc-338 | Santa Cruz |
| SC121 | Human cytosol | Graft human cells | Mouse | 1:500 | AB-121-U-050 | Stem Cells |
| ­SMI-312 | Pan-Axonal Neurofilament Marker | Mature neurons | Mouse | 1:500 | ab24574 | Abcam |
| Synapsin1 | Synapsin 1 | Mature neurons | Rabbit | 1:500 | AB1543 | Millipore |
| Synaptophysin | Synaptophysin (clone EP10) | Mature presynaptic terminals by human specific synaptophysin | Mouse | 1:500 | SMC-178 | StressMarq |
| TBR1 | T-box brain gene 1 | Layer I,V,VI, subplate cortical neurons | Rabbit | 1:200 | ab31940 | Abcam |
| TUJ1 | Neuron-specific Class III β-tubulin | Early neurons | Mouse | 1:500 | T5076 | Sigma |
| TUJ1 | Neuron-specific Class III β-tubulin | Early neurons | Rabbit | 1:500 | PRB-435-P | Covance |
| vGAT | Vesicular GABA transporter | Mature inhibitory GABAergic neurons | Rabbit | 1:200 | 131-103 | Synaptic Systems |
| vGLUT1 | Vesicular glutamate transporter 1 | Mature excitatory glutamatergic neurons | Rabbit | 1:500 | 135-303 | Synaptic Systems |
| vGLUT2 | Vesicular glutamate transporter 2 | Mature excitatory glutamatergic neurons | Rabbit | 1:500 | 135-403 | Synaptic Systems |
